# Biomolecule–Photosensitizer Conjugates: A Strategy to Enhance Selectivity and Therapeutic Efficacy in Photodynamic Therapy

**DOI:** 10.3390/ph19010065

**Published:** 2025-12-29

**Authors:** Dominik M. Płaskonka, Dominik Barczyk, Paweł Repetowski, Marta Warszyńska, Janusz M. Dąbrowski

**Affiliations:** 1 Faculty of Chemistry, Jagiellonian University, 30-387 Kraków, Poland; dominikmarcin.plaskonka@doctoral.uj.edu.pl (D.M.P.); dominik.barczyk@doctoral.uj.edu.pl (D.B.); pawel.repetowski@doctoral.uj.edu.pl (P.R.); m.warszynska@doctoral.uj.edu.pl (M.W.); 2 Doctoral School of Exact and Natural Sciences, Jagiellonian University, 30-348 Kraków, Poland

**Keywords:** antibodies, aptamers, biomolecules, carbohydrates, conjugates, hypoxia, organoids, photodynamic therapy, photosensitizers

## Abstract

Biomolecule–photosensitizer conjugates have rapidly evolved into one of the most powerful strategies for improving the selectivity, efficacy, and translational potential of photodynamic therapy (PDT). By integrating photosensitizers (PSs) with carbohydrates, amino acids, peptides, aptamers, proteins, cofactors, vitamins or antibodies, these constructs overcome long-standing limitations of classical PDT, including poor solubility, insufficient tumour accumulation, and strong dependence on oxygen availability. Beyond enhancing receptor-mediated uptake and enabling precise interactions with the tumour microenvironment (TME), bioconjugation also modulates aggregation, photochemical properties, intracellular accumulation, and immune system activation. A particularly transformative trend is the emergence of supramolecular architectures in which photosensitizers form defined nanostructured aggregates with peptides or proteins. Once considered an undesirable phenomenon, aggregation is now recognized as a tenable feature that governs photochemical behaviour. Engineered aggregates can undergo environment-triggered disassembly to monomeric, photoactive states, or operate as semiconductor-like nanodomains capable of Type I reaction through symmetry-breaking charge separation. This shift toward oxygen-independent radical pathways offers a promising solution to the challenge of hypoxia, a hallmark of the TME that severely compromises conventional Type II PDT. Parallel advances in 3D experimental platforms such as tumour organoids and organ-on-chip systems provide physiologically relevant validation of these conjugates, enabling the assessment of penetration, subcellular localization, immunogenic cell death, and therapeutic synergy within realistic TME conditions. Collectively, the integration of biomolecular targeting with controlled supramolecular design is redefining the landscape of PDT. Future progress will depend on designing conjugates that retain high activity under hypoxia, engineering dynamic aggregate states, and systematically validating these systems in advanced TME-mimetic models. Together, these developments position biomolecule–photosensitizer conjugates as a versatile and increasingly less oxygen-dependent class of next-generation phototherapeutic agents.

## 1. Introduction

Cancer remains one of the most demanding global health problems because malignant tissues continually evolve and adapt under selective pressure. Genomic instability drives the acquisition of key hallmarks, including immune escape, dysregulated proliferation, and aberrant angiogenesis, while sustained growth rapidly disrupts tissue homeostasis. As tumours enlarge, their metabolic needs exceed the available oxygen supply, creating regions of chronic hypoxia. This low-oxygen environment profoundly influences therapy response by diminishing the efficacy of radiotherapy and many chemotherapeutic agents and by favouring the selection of more invasive, treatment-resistant phenotypes.

The combination of persistent hypoxia, metabolic stress, and ongoing genetic diversification produces marked intratumor heterogeneity. Instead of forming a uniform lesion, the tumour becomes a mixture of subclonal populations that differ in genotype, phenotype, and drug sensitivity. Conventional therapies often eradicate dominant clones but fail to eliminate pre-existing resistant variants, which then repopulate the tumour and drive relapse. The coexistence of a protective, hypoxic microenvironment and a diverse, drug-resistant cell population illustrates why many standard treatments show limited durability and underscores the need for therapeutic strategies capable of overcoming these biological barriers [1,2,3].

**Figure 1:**
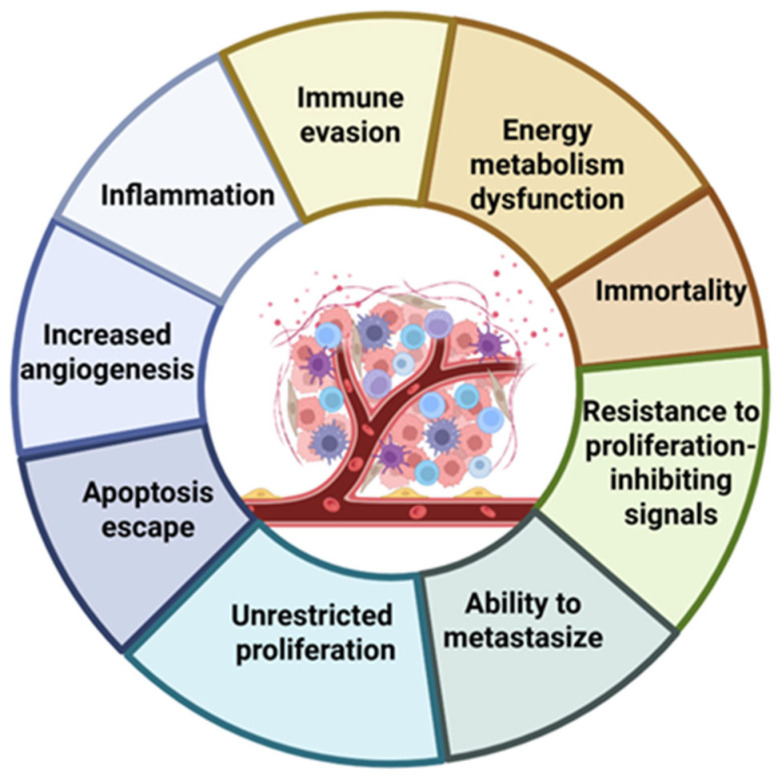
Key biological features of solid tumours contributing to their malignancy that serve as potential targets for biomolecules and biomolecule-photosensitizer conjugates. Representative classes, examples, and cancer models grouped according to the predominant hallmark they target. Created in BioRender. Warszyńska, M. (2025) https://BioRender.com/x0l8b6q (accessed on 18 December 2025).

This set of tumour-associated processes is summarized in Figure 1. The diagram highlights the biological features that drive malignant progression, shape the TME, and contribute to therapeutic resistance. Through the secretion of cytokines, chemokines, and growth factors, tumour cells remodel surrounding tissues, support angiogenesis, and suppress immune activity, processes that collectively enable continued growth and survival. Understanding these interactions is essential for designing therapeutic strategies, including photodynamic therapy-based approaches, that effectively exploit these vulnerabilities. To complement the schematic overview, Table 1 integrates the information from Figure 1 by listing representative biomolecules that exploit these tumour characteristics as therapeutic targets. The table also links each biomolecule to the cancer models in which it has demonstrated activity, illustrating the diversity of biological pathways targeted in contemporary oncology.

PDT represents a mature yet rapidly evolving family of light-driven therapeutic modalities, applied both in oncology [24,25,26,27] and in the management of microbial infections. [28] Recent advances in antimicrobial PDT (aPDT), including the development of tetrapyrrolic and metal-based photosensitizers capable of eradicating bacteria, fungi, and viruses [29], even multidrug-resistant strains in their exponential phase, have demonstrated that appropriately engineered reactive oxygen species (ROS)-generating systems can be both highly potent and intrinsically safe for human tissues [30]. These concepts, supported by our own work on porphyrins [31,32], chlorins [33], bacteriochlorins [30,34], metal complexes [35], and functional materials [36,37,38] for aPDT, provide a framework for translating photodynamic mechanisms into diverse clinical contexts [39].

Building on these foundations, PDT has become an established and increasingly refined therapeutic approach for solid tumours. Its fundamental mechanism relies on the administration of a photosensitizer (PS), followed by illumination with a proper light source matching the PS’s absorption profile. 

**Figure 2:**
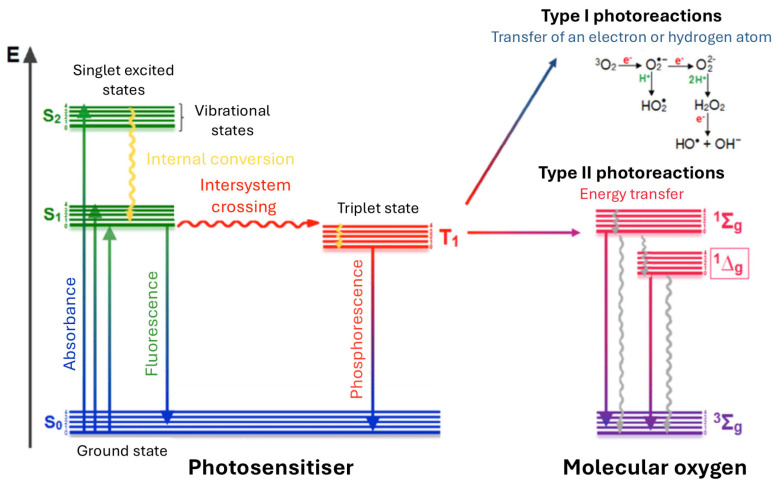
Jablonski diagram showing the electronic states (S_0_, S_1_, S_2_, T_1_) and the main photophysical and photochemical pathways, including absorption, internal conversion, intersystem crossing, fluorescence, phosphorescence, and competing photochemical reactions originating from the triplet excited states.

As illustrated in the Jablonski diagram (Figure 2), photon absorption promotes the photosensitizer from its singlet ground state (S_0_) to short-lived singlet excited states (S_1_, S_2_). The S_2_ state typically relaxes via internal conversion, while the S_1_ state can return to the ground state through radiative fluorescence. The key step for therapeutic efficacy, however, is the transition to the triplet excited state (T_1_) via intersystem crossing (ISC). The T_1_ state is relatively long-lived (microseconds to milliseconds), allowing the PS to interact with surrounding molecules via two competing mechanisms. In Type I reactions, the triplet sensitizer undergoes electron or hydrogen transfer with surrounding substrates, generating radical species and radical ions. These primary radicals can subsequently interact with molecular oxygen, leading to the formation of reactive oxygen species (ROS), including the superoxide ion (O_2_^•−^), and, through secondary reactions, hydrogen peroxide (H_2_O_2_), and hydroxyl radicals (HO^•^). In Type II reactions, energy is transferred directly to molecular oxygen (^3^O_2_), generating highly cytotoxic singlet oxygen (^1^O_2_). Although Type II is often the dominant pathway for many porphyrinoids, the balance between these mechanisms depends on the dioxygen concentration and the local substrate environment. Notably, the photogenerated ROS can also react with the PS itself, leading to photobleaching (photodegradation), which may limit the effective therapeutic dose over time [40].

The photochemical behaviour of PSs in their triplet excited states is governed by the interplay between diffusive energy transfer, charge-transfer (CT) processes, and the intrinsic redox properties of the given PS [41,42,43]. Upon population of the triplet excited state, the PS can undergo deactivation via competing pathways that determine both the kinetics of quenching and the distribution of generated ROS [44,45]. In the absence of significant contribution from CT pathway, quenching is diffusion-limited, resulting in efficient energy transfer to molecular oxygen and near-unity quantum yields of ^1^O_2_ generation [41,44]. When CT interactions are operative, the triplet state can engage in partial electron or hole transfer processes involving molecular oxygen, accelerating the overall quenching rate. This increased rate of triplet deactivation reduces the yield of singlet oxygen, as a portion of the triplet energy is diverted into alternative ROS-generating channels, such as superoxide ion and eventually hydroxyl radicals. The propensity for CT-mediated quenching is strongly influenced by the redox potential of the PS: a lower oxidation potential facilitates electron transfer processes, thereby enhancing CT pathways and the formation of oxygen-centered radical species, whereas a higher oxidation potential can suppress CT pathways and favour diffusion-limited singlet oxygen generation. Critically, the redox potential also governs photostability, as excessively low oxidation potentials can render the PS susceptible to self-oxidation and photodegradation, while appropriately balanced potentials preserve the integrity of the molecule during repeated excitation cycles [45]. This inverse correlation between the quenching rate and ^1^O_2_ quantum yield constitutes a fundamental mechanistic signature of CT-mediated photochemistry [41,46,47].

**Figure 3:**
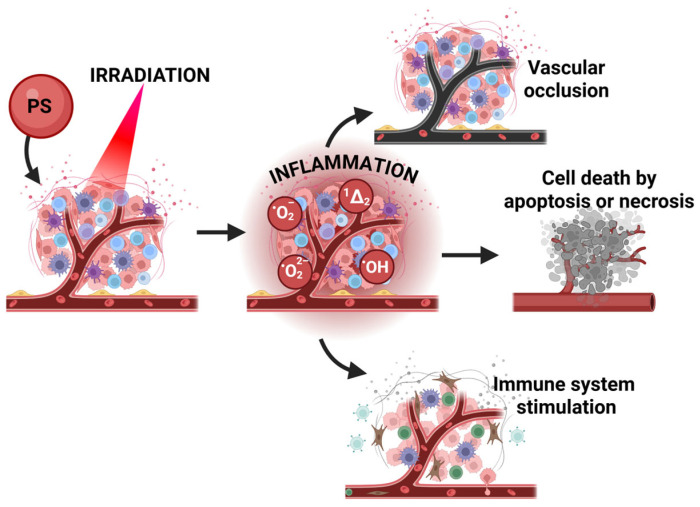
Effects of PDT occur after irradiation of the tumour, when the photosensitizer (PS) is present and is promoted to its triplet excited state, leading to the generation of ROS. The resulting oxidative stress induces vascular occlusion, cancer cell death, and activation of antitumour immune responses. Created in BioRender. Warszyńska, M. (2025) https://BioRender.com/dc9o5a6 (accessed on 18 December 2025).

The relative contributions of CT and non-CT pathways are thus central to determining both the lifetime of the triplet excited state and the composition of the resulting ROS generation. Fine-tuning the electronic and redox properties of the PS modulates these competing channels, controlling the balance between fast quenching, singlet oxygen production, alternative ROS generation, and photostability. Understanding this balance provides a predictive framework for the rational design of photosensitizers, wherein the combined manipulation of CT dynamics and redox potential allows selective favouring of specific ROS pathways while ensuring optimal (photo)stability and desired photochemical performance [30,47,48]. However, the ultimate effect of ROS on tumour strongly depends not only on the photophysical properties but also on PS structure (which further influences its pharmacological parameters), time between administration and irradiation, a so-called drug-to-light interval (DLI), and formulation used [49,50,51,52]. Oxidative stress generated by ROS induces local acute inflammation leading to cell death through multiple pathways, most commonly apoptosis and necrosis, as well as vascular occlusion, which subsequently activates the immune system (Figure 3). Despite its broad clinical relevance, many conventional PSs display inherent limitations, including poor aqueous solubility, insufficient selectivity toward diseased tissues, and inadequate accumulation within the tumour [53,54]. These drawbacks substantially restrict both therapeutic efficacy and clinical translation.

To address these limitations, recent research has increasingly focused on conjugating PSs with biologically active molecules (such as amino acids, peptides, proteins, antibodies, cofactors, vitamins, and carbohydrates), aiming to improve their physicochemical and pharmacological profiles [55,56]. Bioconjugation strategies have demonstrated the capacity to enhance solubility and bioavailability while enabling targeted accumulation in tumour tissues through mechanisms such as the enhanced permeability and retention (EPR) effect and receptor-mediated internalization [57,58]. Furthermore, the incorporation of biomolecular ligands facilitates selective interactions with tumour-associated biomarkers and receptors present within the tumour microenvironment [59]. In addition, PS-biomolecule conjugates enable more effective engagement with complex cellular microenvironments, including chemokine-rich niches expressing receptors such as CCR5 and CCR7 [60], which modulate tumour invasiveness and immune cell infiltration [61]. Consequently, these next-generation conjugated PSs represent a promising class of phototherapeutic agents capable of integrating molecular specificity with high phototoxic efficiency [62,63].

Beyond classical receptor targeting, an increasingly important aspect of biomolecule–photosensitizer conjugates is their ability to self-assemble into supramolecular nanostructures once introduced to aqueous or physiological environments. Many peptide- and protein-based conjugates spontaneously form well-defined aggregates that protect the chromophore, modulate its photophysical properties, and influence the fate of the construct in vivo. Although aggregation has traditionally been recognized as detrimental because it may quench PS’s excited states or suppress singlet oxygen generation, growing evidence shows that this assumption is overly simplified. Numerous studies demonstrate that aggregated tetrapyrroles, including porphyrins, chlorins, and phthalocyanines, can remain photodynamically active and, in some cases, exhibit enhanced photothermal or photochemical responses within crowded biological media.

Equally important is the observation that these assemblies rarely remain static. Interactions with lipids, serum proteins, extracellular matrix components, or intracellular targets can trigger partial or complete monomerization, resulting in a conditional “activation” of the PS. Such transitions are particularly relevant for tumour tissue, where gradients of pH, ionic strength, enzymatic activity, and membrane composition create a heterogeneous landscape that selectively destabilizes aggregates. In this way, biomolecular conjugation does not merely improve solubility or targeting; it introduces a second regulatory layer in which assembly and disassembly govern biodistribution, subcellular localization, and the balance between photodynamic and photothermal mechanisms.

These concepts collectively position biomolecule–photosensitizer conjugates as versatile platforms capable of exploiting the physicochemical complexity of the TME. By integrating molecular recognition, controlled supramolecular organization, and light-triggered cytotoxicity, they offer a route toward more selective and adaptive photodynamic therapies. The following sections examine the major classes of biomolecules used for conjugation, the structural principles that govern their assembly and biological behaviour, and the therapeutic opportunities emerging from these hybrid systems. Because their supramolecular behaviour is strongly modulated by the biochemical landscape of the TME, these conjugates are uniquely positioned to exploit TME-specific cues for targeted activation and delivery.

## 2. The Tumour Microenvironment as a Target for Photosensitizer Delivery

Tumour microenvironment is a highly dynamic and heterogeneous biological system that consists of malignant cells, stromal fibroblasts, endothelial cells, immune cell populations, and the extracellular matrix (ECM). These components interact through complex signalling networks composed of cytokines, chemokines, and growth factors. Importantly, the TME displays several physicochemical abnormalities (hypoxia, acidic extracellular pH, elevated interstitial pressure, and irregular vasculature) that collectively impair drug delivery, including the transport and accumulation of photosensitizers [64].

These structural and biochemical features of solid tumours strongly influence the distribution of PSs and the treatment outcome of PDT. Disorganized and leaky tumour vasculature typically results in heterogeneous blood flow, while high interstitial pressure impedes diffusion of therapeutic agents, ultimately reducing PS penetration into tumour tissue [65]. Hypoxia is particularly detrimental, as it limits the generation of singlet oxygen, one of the key cytotoxic agents in PDT [66]. Tumour hypoxia arises not only from inadequate vascular density but also from profound abnormalities in tumour perfusion and oxygen transport. The highly permeable vasculature characteristic of solid tumours leads to heterogeneous blood flow, resulting in regions of transient (perfusion-limited) and chronic (diffusion-limited) hypoxia. Even when blood vessels are present, steep oxygen diffusion gradients develop as oxygen is rapidly consumed by metabolically active cancer cells, causing a sharp decline in the partial pressure of oxygen (pO_2_) with increasing distance from functional capillaries. Consequently, pO_2_ values in tumours often fall well below those of normal tissues, frequently reaching levels insufficient to sustain efficient Type II photochemical reactions. Reduced pO_2_ therefore directly limits ^1^O_2_ production and diminishes PDT efficacy, while favouring the survival of hypoxia-adapted tumour cell populations [67,68].

Importantly, tumour hypoxia should be considered a dynamic parameter that evolves before, during, and after photodynamic treatment, making pO_2_ monitoring essential for accurate evaluation of treatment response. Several studies have shown that vascular-targeted PDT can induce extremely low and sustained pO_2_ levels lasting several days, which correlates with durable tumour control, whereas tumour cell-targeted PDT typically produces only mild and transient hypoxia, often followed by compensatory reoxygenation and tumour regrowth. These observations demonstrate that the temporal profile and severity of PDT-induced hypoxia, rather than hypoxia alone, critically determine therapeutic outcome [69,70].

In parallel, hypoxia is closely associated with extracellular acidosis, driven by glycolytic metabolism and poor clearance of acidic byproducts. The resulting low pH in the TME profoundly influences the physicochemical behaviour of photosensitizers and their delivery systems [71,72]. Acidic conditions can promote aggregation of hydrophobic molecules, quenching PS’s excited states and reducing ROS generation efficacy, while also altering their charge state, cellular uptake, and subcellular localization. Conversely, pH gradients can be exploited to enhance PDT selectivity, as protonation under acidic conditions may increase the retention of cationic PSs in tumour cells or trigger the disassembly of pH-responsive nanocarriers, leading to localized release of the PS [73,74]. Moreover, pH-dependent changes in protein conformation and carrier stability can modulate PS–protein interactions, circulation time, and tumour accumulation [75]. Together, the interplay between abnormal perfusion, oxygen diffusion gradients, low pO_2_, and acidic pH defines a complex microenvironment that not only constrains conventional Type II-PDT but also motivates the development of hypoxia- and pH-adaptive photosensitizers and carrier systems capable of maintaining therapeutic efficacy under the harsh conditions of solid tumours [76].

To mitigate the limitations imposed by low pO_2_, increasing emphasis has been placed on PSs and supramolecular systems that operate via less oxygen-dependent mechanisms. In particular, PSs engaging Type I photochemical pathways generate cytotoxic radical species, thereby retaining activity under hypoxic conditions where singlet oxygen production is severely compromised [77,78,79]. Complementary approaches exploit hypoxia-responsive delivery systems that undergo selective activation or enhanced intracellular release in oxygen-depleted TME. Together, these strategies reframe hypoxia from a purely inhibitory factor into a design parameter for engineering adaptive photoactivated systems capable of sustaining PDT efficacy in solid tumours [80,81]. In this context, clinically relevant photosensitizers such as padeliporfin (WST11, Tookad^®^) and redaporfin have been highlighted for their pronounced engagement of Type I photochemical pathway, vascular targeting, and sustained photodynamic efficacy [3]. In our lab, we have recently developed 5,10,15,20-tetrakis-[2′,3′,5′,6′-tetrafluoro-4′-methanesulfamoyl)phenyl] bacteriochlorin (F_4_BMet), a PS that acts via both Type I and Type II mechanisms, which can be used for the treatment of hypoxic tumours [82]. To overcome these delivery barriers, several strategies targeting the TME have been developed. Passive targeting, which exploits EPR, enables nanoscale PS formulations to accumulate selectively within the TME. However, because EPR is highly variable between types of cancer and even within individual tumours, active targeting has gained substantial attention. This approach uses ligands (peptides, antibodies, carbohydrates, or small molecules) that recognize overexpressed receptors in tumour cells or stromal elements. Hypoxia-responsive nanomaterials represent a major class of TME-targeting systems. One prominent example is a supramolecular hypoxia-modulating nanoplatform based on pillar arene that co-delivers a cyanine-based PS and atovaquone [5]. In the acidic TME, this platform disassembles, releasing atovaquone to inhibit mitochondrial respiration and thereby conserve oxygen, significantly enhancing PDT efficacy [83]. Similarly, MnO_2_-based nanostructures have been utilized as smart oxygen-generating carriers: in acidic and reductive tumour regions, MnO_2_ decomposes endogenous H_2_O_2_ to generate O_2_, improving PDT outcomes and enabling multimodal imaging [65,84,85].

Another promising direction is photodynamic therapy integrated with immunotherapy. PDT can induce immunogenic cell death (ICD), which stimulates dendritic cells activation and promotes antitumour immune responses. Nanoplatforms that combine PSs with immunostimulatory molecules or oxygen-generating components have been shown to enhance ICD and synergize with checkpoint blockade therapies [64,86]. A closely related and rapidly expanding field is photoimmunotherapy (PIT), in which antibodies are conjugated to PSs such as IR700. This enables highly specific, ligand-mediated PS delivery and rapid cell death upon near-infrared activation. While PIT was initially focused on tumour-cell antigens (e.g., EGFR or HER2), recent studies demonstrate that targeting stromal components, including cancer-associated fibroblasts or immunosuppressive regulatory T cells, enhances therapeutic efficacy by restructuring the TME itself [87,88,89,90,91,92,93].

Finally, TME-normalizing strategies, such as vascular modulation or ECM-remodelling nanoparticles, have shown the ability to improve perfusion and decrease hypoxia, thereby facilitating PS accumulation into the tumour and heightened PDT efficacy. Such approaches are particularly promising when integrated into multifunctional nanoplatforms that simultaneously target multiple barriers within the TME [66]. Collectively, these advances demonstrate that the biochemical and structural heterogeneity of the TME is not only an obstacle but also a valuable therapeutic target. Rationally engineered TME-responsive PS delivery systems hold the potential to significantly increase tumour selectivity, penetration, and overall photodynamic efficacy.

To fully realize the therapeutic potential of these TME-responsive delivery systems, their biological performance must be rigorously assessed across cellular, three-dimensional, and in vivo models. This requires a systematic evaluation of whether biomolecule–photosensitizer conjugates retain target specificity, photochemical competence, and favourable pharmacological behaviour within complex biological environments.

## 3. Development of Biomolecule–PS Conjugates

The design of new conjugates is based primarily on selected characteristics of tumors and the tumour microenvironment, rather than on the photophysical and photochemical properties of classical photosensitizers. This approach considers factors such as tumour-specific metabolism, hypoxia, aberrant tumour vasculature, and the presence of distinct cellular populations within the TME. By focusing on these biological features, newly developed conjugates can achieve improved selectivity, enhanced accumulation at the tumour site, and more effective therapeutic responses compared to traditional PSs whose development relies mainly on their intrinsic optical and photochemical properties.

**Figure 4:**
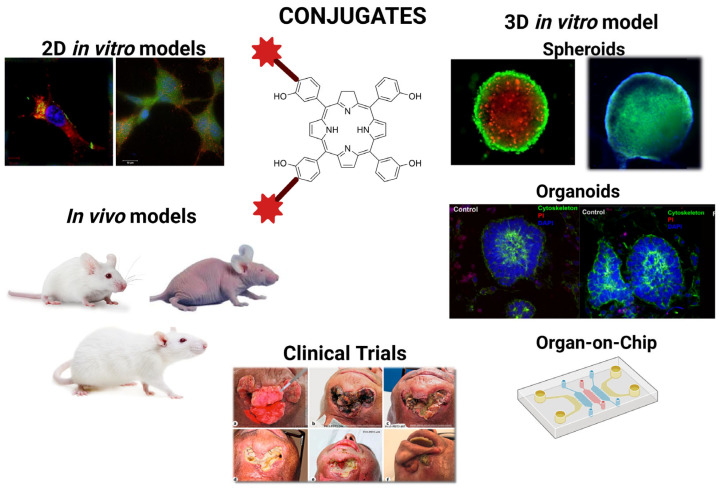
Different models used for the biological activity assessment of new conjugates: 2D in vitro cancer cell lines, 3D in vitro models: spheroids, organoids, and organ-on-chip, in vivo animal models, and lastly clinical trials on patients. Adapted from [82,96,97] and modified. Created in BioRender. Warszyńska, M. (2025) https://BioRender.com/vf84taj (accessed on 18 December 2025).

The evaluation of the biological activity of biomolecule–PS conjugates represents a crucial stage in their preclinical development, as it determines whether such constructs can deliver the desired combination of target selectivity and improved efficacy. In vitro and in vivo investigations must therefore be integrated to assess target recognition, cellular uptake, and subcellular distribution, photochemical performance in the cellular environment (including ROS detection), toxicity without irradiation, light-dependent cytotoxicity, pharmacokinetics, biodistribution, and safety (Figure 4). A comprehensive assessment across these dimensions enables the selection of lead conjugates with favourable therapeutic indices and reasonable translational potential [94,95]. Because many conjugates form supramolecular assemblies that undergo partial disassembly in cellular or tumour-associated environments, the evaluation of biological activity must also consider changes in aggregation state and their impact on photophysical behaviour and intracellular routing.

Determination of targeting specificity begins with quantitative binding studies and competitive assays that compare uptake in cells with high versus low expression of the intended molecular target. Flow cytometry, quantitative fluorescence imaging, and ligand-blocking experiments provide robust characterization of selective accumulation, while biochemical assays can establish whether conjugation chemistry has altered the intrinsic affinity of the biomolecular targeting moiety. It is important to demonstrate that the process of linking a PS preserves the target-recognition properties of the biomolecule, because even modest changes in affinity or steric presentation can substantially change tumour targeting [98,99]. This is particularly relevant for conjugates that rely on multivalent or peptide-based assemblies, in which even subtle modifications of surface charge or steric presentation may alter receptor engagement and downstream internalization pathways.

Photophysical and photochemical evaluation of the conjugate is required to confirm that the resulted photomaterial retains efficient light absorption and productive energy and/or electron transfer after conjugation. Standardized assays for ROS detection, including specific singlet oxygen probes and general ROS-sensitive dyes, should be accompanied by measurements of singlet oxygen quantum yield and photostability under clinically relevant irradiation conditions. These experiments not only determine the intrinsic photodynamic potential of the material, but also specify dosing and light-delivery strategies, since reduced quantum yields or rapid photobleaching will adversely affect photodynamic effectivity and reproducibility [94,100]. For conjugates that form nanoaggregates in aqueous or physiological media, photophysical and photochemical studies should include a comparison of monomeric and aggregated states, since supramolecular organization can suppress, enhance, or shift ROS generation pathways.

In vitro photocytotoxicity testing provides the first functional readout of therapeutic activity and requires careful distinction between light-activated and dark toxicity. Cell viability assays performed across a range of conjugate concentrations and light doses, together with dark-control conditions, quantify the light-to-dark toxicity ratio (a key parameter for assessing selectivity). Mechanistic studies using apoptosis and necrosis markers, caspase activation, and assays of mitochondrial membrane potential help to define the dominant cell death pathway elicited by the conjugate. These readouts are also shaped by the kinetics of intracellular disassembly, particularly in conjugates designed to remain aggregated during uptake but release monomeric photosensitizers upon interaction with lipids, proteases, or acidic compartments. Such mechanistic fingerprints are influenced strongly by the subcellular localization of the studied materials [95,98].

Subcellular localization studies are therefore central to mechanistic interpretation: PSs that accumulate in mitochondria typically promote apoptotic cascades, whereas localization in lysosomes can provoke lysosomal disruption and secondary cell death mechanisms; endoplasmic reticulum (ER) localization may cause rapid necrotic or immunogenic responses in the form of immunogenic cell death. Fluorescence confocal microscopy using organelle-specific markers, fractionation followed by quantitative analysis, and live-cell imaging under irradiation conditions yield complementary information that connects intracellular distribution to observed cytotoxic outcomes. Biomolecular carriers such as cell-accumulating peptides, receptor-targeting aptamers, and affibodies have been shown to substantially bias intracellular routing and thereby alter the biological response to light activation [99,101]. In conjugates that undergo supramolecular reorganization inside cells, organelle-specific trafficking often depends not only on the targeting ligand but also on aggregate size, surface chemistry, and the rate of intracellular de-aggregation.

In the evaluation of biological activity, three-dimensional in vitro models have become increasingly important for assessing the efficacy of biomolecule–PS conjugates, as they more accurately replicate the native tissue microenvironment than conventional two-dimensional cultures [15]. Key models include spheroids, organoids, and organ-on-chip (OoC) systems. Spheroids provide compact cellular aggregates with oxygen and nutrient gradients, as well as proliferative and necrotic zones, offering a physiologically relevant context to evaluate cellular uptake and photodynamic effects. Organoids, derived from stem cells or primary tissues, recapitulate tissue architecture and functionality, allowing the investigation of conjugate penetration, subcellular localization, and phototoxicity in complex 3D structures. Integration with microfluidic OoC platforms enables precise modulation of the microenvironment and real-time monitoring of therapeutic efficacy [82,102]. Despite their predictive value, 3D models present challenges, including variability between organoid batches and the technical complexity of OoC systems [102]. Accordingly, the careful selection of the appropriate 3D platform and consideration of its limitations are critical for rigorous evaluation of biomolecule–PS conjugates. These platforms are particularly informative for conjugates that display pH-dependent or protein-induced disassembly, as gradients of oxygenation, acidity, and matrix density in 3D culture better reproduce the cues that regulate supramolecular behaviour in vivo.

Brain organoid platforms provide a physiologically relevant system to evaluate conjugates, for example, in the context of 5-ALA–based PDT. 5-ALA is a prodrug that is metabolized into protoporphyrin IX (PpIX), and due to the decreased activity of ferrochelatase in tumour cells, it does not convert further into heme. PpIX accumulates in tumour cells, exhibiting strong phototherapeutic efficacy and red fluorescence, making it a useful tool both in PDT and fluorescence imaging [103]. GBM–cerebral organoid co-cultures recapitulate tumour heterogeneity, metabolic gradients, and invasive behaviour, enabling detailed assessment of PpIX accumulation, tissue penetration, and light-induced cytotoxicity. Upon irradiation, 5-ALA–derived PpIX triggers apoptosis via mitochondrial caspase activation and ROS-mediated stress, while sparing non-malignant tissue. These in vitro observations demonstrate selective and well-tolerated PDT effects [104,105]. Although 5-ALA operates through endogenous biosynthesis rather than covalent conjugation, the selective accumulation of PpIX illustrates how microenvironment-dependent enzymatic processes can mimic features of stimulus-responsive PS delivery.

In our laboratory, we have used colorectal cancer (CRC) organoids from human induced pluripotent stem cells (hiPSC) to confirm the biological activity of recently developed photosensitizers [82,103]. Dark cytotoxicity and photodynamic efficacy of PSs have been evaluated against CRC organoids, presenting a high phototoxic effect, and safety without irradiation. 3D model validated result obtained with classical in vitro models. The minimal aggregation of PS in biological media, resulting from either perfluorinated aryl substituents or tert-butylsulfonyl substituents, further illustrates how rational molecular design can modulate supramolecular organization and improve consistency of photodynamic response in complex models.

For bladder cancer, patient-derived organoids containing tumour epithelium, fibroblasts, and smooth-muscle cells have been used to evaluate combination therapies such as ionizing radiation plus PDT. These models reveal synergistic cytotoxicity via apoptosis, necroptosis, ferroptosis, and pyroptosis, while promoting immune cell infiltration [106].

Photosensitizers such as phthalocyanines and 2,3-naphthalocyanine are promising for conjugation due to strong NIR absorption and efficient ROS generation, but their aggregation, hydrophobicity, and limited tumour uptake pose challenges. Their tendency to form tightly packed aggregates in aqueous environments highlights why evaluation must consider supramolecular behaviour, as aggregation can profoundly alter ROS profiles and intracellular fate. The biomolecule-conjugation strategy improves solubility, receptor-mediated uptake, and subcellular targeting. Organoid studies demonstrate that cyanine and phthalocyanine PS exhibit enhanced photodynamic activity in 3D systems when intracellular routing is optimized via biomolecular carriers [107,108].

Organoid platforms are also crucial for evaluating PDT combined with chemotherapy. By preserving tumour-specific ECM, stromal interactions, and diffusion barriers, organoids enable realistic assessment of drug-PS co-delivery, synergy, and resistance mechanisms. Incorporating biomolecule–PS conjugates, including our NIR-PS, expands the therapeutic window by increasing tumour selectivity and minimizing off-target toxicity [82].

Therapeutic efficacy in animal models should be evaluated with attention to clinically meaningful endpoints. In solid tumour models, demonstration of tumour-growth delay, objective tumour regression, and survival benefit compared with free photosensitizer controls are minimal requirements. Moreover, because PDT can induce ICD, studies evaluating adaptive and innate immune responses, such as dendritic cell maturation, tumour-infiltrating lymphocyte profiles, distant (abscopal) effects, and cytokine release, provide valuable information on possible combination strategies with immunotherapies. Targeted constructs based on affibodies or antibodies have shown promising tumour specificity and immune-modulatory effects in several preclinical studies, highlighting the translational potential of biomolecule–photosensitizer conjugates [101,109].

Finally, an explicit appraisal of safety, skin photosensitivity, and immunogenicity must be performed before clinical translation is contemplated. For conjugates that exist in aggregated form during circulation, safety assessment must also consider whether nanoparticle size, protein adsorption, or aggregation-driven pharmacokinetics influence off-target accumulation or immunogenicity. Hematotoxicity and cytotoxicity toward primary, non-transformed cells, genotoxicity screens where appropriate, and assessment of any anti-drug antibody responses are required to establish a tolerable therapeutic index. When conjugates are advanced to formal preclinical development, regulatory expectations also demand standard pharmacology and toxicology packages that are aligned with the intended clinical use and route of administration. Taken together, rigorous, hypothesis-driven evaluation of biodistribution, mechanism of action, efficacy, and safety is required to advance biomolecule–photosensitizer conjugates from promising preclinical constructs to clinically viable PDT agents [94,95]. Overall, the integration of classical biological assays with analyses of supramolecular behaviour provides a more complete framework for prioritizing conjugates with predictable in vivo performance.

Table 2 summarizes the principal experimental models used to evaluate the biological activity of biomolecule–PS conjugates applied in PDT. The table includes a concise characterization of each model type, methodological relevance, and typical endpoints used to assess targeting selectivity, photochemical performance, cytotoxic mechanisms, biodistribution, therapeutic efficacy, and safety.

## 4. Types of Biomolecules Used for Photosensitizer Modification

The conjugation of photosensitizers with biomolecules represents a powerful approach to improving their physicochemical and biological performance in PDT. Each class of biomolecule: carbohydrates, aptamers, cofactors, vitamins, amino acids, peptides, and proteins, or more specifically antibodies, provides distinct advantages in terms of solubility, target selectivity, and bioavailability. The selection of the biomolecular moiety is therefore crucial for optimizing the therapeutic outcome and determining the mode of cellular uptake and biodistribution. In addition, the chosen biomolecular scaffold exerts a decisive influence on the supramolecular organization of the conjugate, modulating its aggregation state, photophysical behaviour, and, ultimately, the balance between photodynamic and photothermal mechanisms in complex biological environments.

### 4.1. Carbohydrates

**Figure 5:**
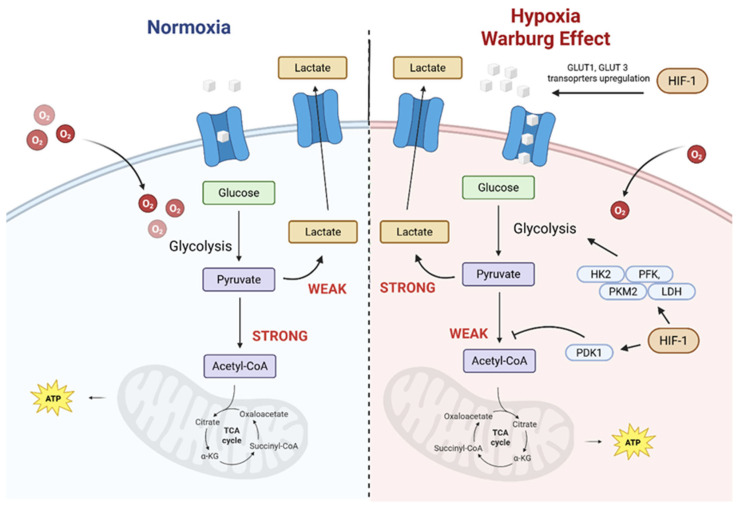
The Warburg effect in cancer cells. Cancer cells exhibit elevated glucose uptake and rely mainly on glycolysis for energy production, even under oxygen-rich conditions. Hypoxia-inducible factor 1 (HIF-1) enhances this process by upregulating glucose transporters (GLUT1, GLUT3) and glycolytic enzymes (HK2, PFK, PKM2, LDH), while inhibiting pyruvate conversion to acetyl-CoA through PDK1 activation. Created in BioRender. Warszyńska, M. (2025) https://BioRender.com/vik2700 (accessed on 18 December 2025).

Carbohydrate conjugation enhances the water solubility of photosensitizers and enables the exploitation of glucose transporters (GLUTs), which are overexpressed in many cancer cells as a result of their elevated glycolytic activity (the Warburg effect, Figure 5) [114,115]. Incorporation of glucose, galactose, or mannose residues facilitates preferential accumulation of the conjugates in metabolically active tumour regions.

Glycoconjugate PSs exhibit improved tumour selectivity and retention compared with their unconjugated analogues. The glycosylation of PS also increases their hydrophilicity, facilitates more precise tumour targeting, mitigates off-target toxicity, and contributes to broader and more favourable pharmacokinetic profiles [114,116,117,118]. Mechanistically, glucose- or galactose-modified porphyrins, chlorins, and phthalocyanines have been demonstrated to enter cells via GLUT-mediated pathways or other carbohydrate-recognition mechanisms; this receptor/transport-assisted internalization generally results in higher cellular uptake and greater phototoxicity in vitro and in vivo than the parent PS (examples include glycosylated chlorins and glycosylated zinc-phthalocyanine derivatives) [119].

Beyond targeting, the hydrophilic carbohydrate moiety plays a critical physicochemical role: it improves aqueous solubility and suppresses π–π stacking/aggregation that commonly plagues hydrophobic macrocyclic photosensitizers. By stabilizing the PS in its active monomeric form in physiological media, glycosylation preserves photophysical properties required for efficient singlet oxygen generation and reasonable fluorescence. Both are key for therapeutic potency and imaging-guided PDT [40,120,121,122].

The cumulative effect of these changes is a favourable shift in pharmacokinetics and biodistribution: increased tumour retention, reduced nonspecific accumulation in healthy tissues, and lower off-target phototoxicity. Several recent preclinical reports and reviews note improved circulation profiles and enhanced tumour-to-normal tissue ratios for well-designed glycoconjugates, especially when the carbohydrate is presented in multivalent or sterically optimized formats [123,124].

A representative example of this design paradigm is the glucose–Zn(II) phthalocyanine conjugate previously reported as a second-generation photosensitizer for tumour-selective PDT. The incorporation of glucose exploits the elevated glucose uptake pathways characteristic of many malignancies, promoting preferential intracellular accumulation of the photosensitizer. At the same time, the hydrophilic sugar unit mitigates aggregation of the phthalocyanine macrocycle, thereby maintaining its monomeric state and ensuring efficient singlet oxygen generation upon irradiation. The conjugate exhibits minimal dark toxicity but retains high photodynamic efficacy, illustrating how carbohydrate functionalization can simultaneously enhance molecular targeting and photochemical performance [125].

A complementary approach arises from a galactose-functionalized BODIPY system developed for NIR-II fluorescence-guided photothermal therapy. The galactose moiety enables selective recognition by hepatocellular carcinoma cells while also stabilizing hydrogen-bonded J-aggregates, shifting absorption and emission into the NIR-II region (1000–1700 nm). This supramolecular organization yields high photothermal conversion efficiency and enables deep-tissue optical imaging coupled with effective thermal ablation. As with other carbohydrate-photosensitizer conjugates, the integration of a sugar unit enhances solubility, reduces nonspecific interactions, and improves targeted delivery, underscoring the broad utility of glycosylation in the design of next-generation precision theranostic agents [126]. Similar results were obtained after conjugation of BODIPY with the mannose moiety [127]. Taken together, these examples illustrate that glycosylation can either stabilize monomeric photosensitizers or promote the formation of well-defined functional aggregates, with both regimes being exploitable for improved PDT or PTT depending on the desired mechanism of action.

**Figure 6:**
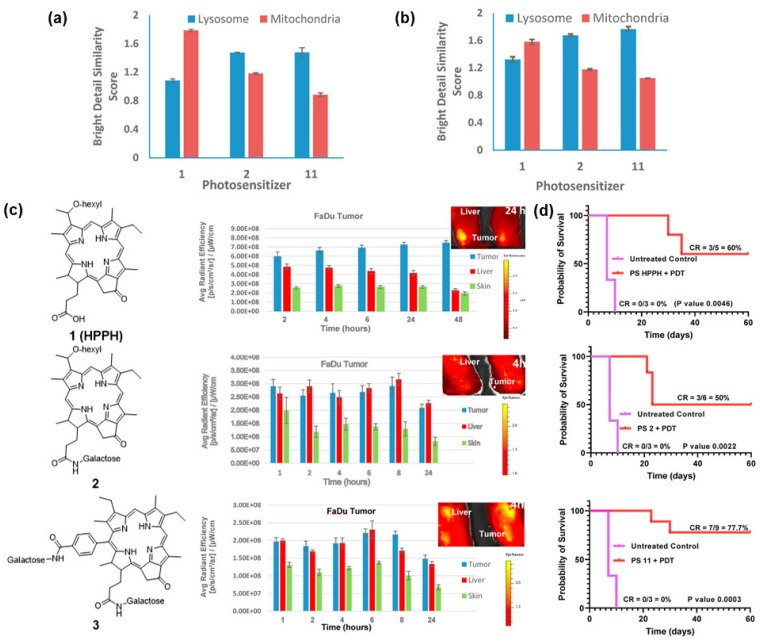
Overview of galactose-pyropheophorbide conjugates for carbohydrate-enhanced PDT. (**a**,**b**) Localization of photosensitizers 1, 2, and 11 in (**a**) FaDu and (**b**) CT26 cells. (**c**) The cellular uptake of the photosensitizers was examined in FaDu tumour-bearing SCID mice following injection of compounds 1 (HPPH), 2, and 3 at a dose of 0.47 μmol/kg (three animals per group), and monitored over a 24-h period. (**d**) Survival rates of SCID mice bearing FaDu tumour. Mice were treated with compounds 1 (HPPH), 2, and 11. Adapted from [128,129] and modified.

Compounds 1 and 2 from Figure 6, representing mono-β-galactose pyropheophorbide conjugates, showed moderate cellular uptake, with compound 2 being internalized more efficiently due to its β-orientation, which better matches galactose-binding proteins expressed on FaDu and colon cancer cells (CT26). In contrast, the di-β-galactose conjugate 11 exhibited markedly higher uptake, reflecting enhanced interactions with galectin-type receptors and altered physicochemical properties that promoted endosomal-lysosomal accumulation. In vivo, compounds 1 and 2 displayed only modest tumour retention and produced limited therapeutic benefit in the xenograft model. The strongest response was achieved with compound 3, which demonstrated favourable biodistribution, prolonged tumour accumulation, and significantly improved PDT efficacy. Following light irradiation, approximately 78% of treated mice remained tumour-free for 60 days, making compound 11 the most effective of the evaluated galactose-modified pyropheophorbides [128].

**Figure 7:**
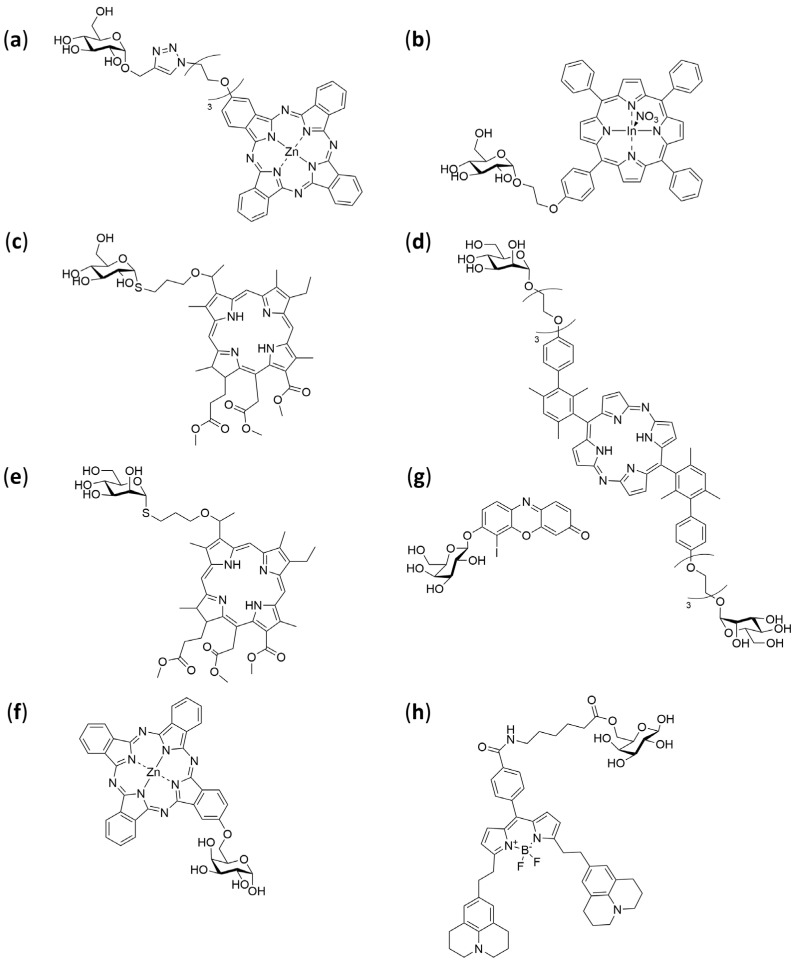
Representative structures of carbohydrate–photosensitizer conjugates (**a**) Zn(II) phthalocyanine–glucose conjugate; (**b**) Indium(III) 5,10,15,20-triphenylporphyrin nitrate–glucose conjugate; (**c**) Chlorin e6–glucose conjugate; (**d**) 5,15-diazaporphyrin–mannose conjugate; (**e**) Chlorin e6–mannose conjugate; (**f**) Zn(II) phthalocyanine–galactose conjugate; (**g**) 7-hydroxy-6-iodo-3H-phenoxazin-3-one–galactose conjugate; (**h**) BODIPY–galactose conjugate.

Taken together, the available literature supports glycosylation as a robust and general strategy to bias cellular uptake toward tumour cells via metabolic and lectin pathways, preserve the monomeric, photoactive state of the PS in aqueous media, and improve the therapeutic index of PDT through more favourable pharmacokinetics and reduced off-target effects. Continued optimization of linker chemistry, sugar type/valency, and site of attachment remains important for translating these advantages into clinically useful PS constructs [114,119,123]. Table 3 summarizes notable carbohydrate–PS conjugates reported to date, together with the experimental models employed to evaluate their photophysical properties, biological activity, and therapeutic potential. Moreover, Figure 7 presents examples of various PSs conjugated with carbohydrates.

### 4.2. Aptamers

Nucleic acid aptamers represent a highly versatile and distinct class of targeting ligands for the delivery of various photosensitizers. Often termed “chemical antibodies,” aptamers are short, single-stranded DNA or RNA oligonucleotides (typically 20–80 nucleotides) selected from massive combinatorial libraries via a process known as SELEX (Systematic Evolution of Ligands by Exponential Enrichment) [136,137,138,139]. Unlike linear genetic material used for coding, aptamers are designed to fold into unique tertiary structures, such as hairpins, pseudoknots, or G-quadruplexes, which allow them to bind to specific molecular targets with high affinity and specificity [140]. The conjugation of PSs to aptamers offers several structural and therapeutic advantages, primarily by transforming hydrophobic, non-specific PSs into hydrophilic, targeted therapeutic agents (Table 4). Addressing the challenge of PS aggregation, recent research has demonstrated the potential of molecularly engineering the aptamer sequence itself to function as a solubilizer. Instead of relying on complex nanocarriers, specific aptamer designs have been shown to interact non-covalently with hydrophobic NIR photosensitizers via intercalation. This approach creates a simplified, highly efficient “self-delivery” system that ensures deep tissue penetration and improved bioavailability without compromising photodynamic efficacy [129].

Specifically, this strategy was successfully applied to solubilize the hydrophobic near-infrared PS, pyropheophorbide a (PA), by leveraging the Sgc8 aptamer, which exhibits high affinity for the protein tyrosine kinase 7 (PTK7) receptor. By employing automated DNA solid-phase synthesis combined with Cu-free click chemistry, the researchers achieved precise loading of the PS onto the oligonucleotide backbone. This modification effectively transformed the insoluble dye into a highly hydrophilic agent (solubility ~750 µM) without requiring surfactant co-solvents or auxiliary nanocarriers. For in vivo applications, such conjugates typically require additional chemical stabilization of the oligonucleotide backbone (for example, 2′-modified nucleotides, PEGylation, or phosphorothioate linkages) to prolong circulation time and mitigate nuclease-mediated degradation, without compromising target affinity.

**Figure 8:**
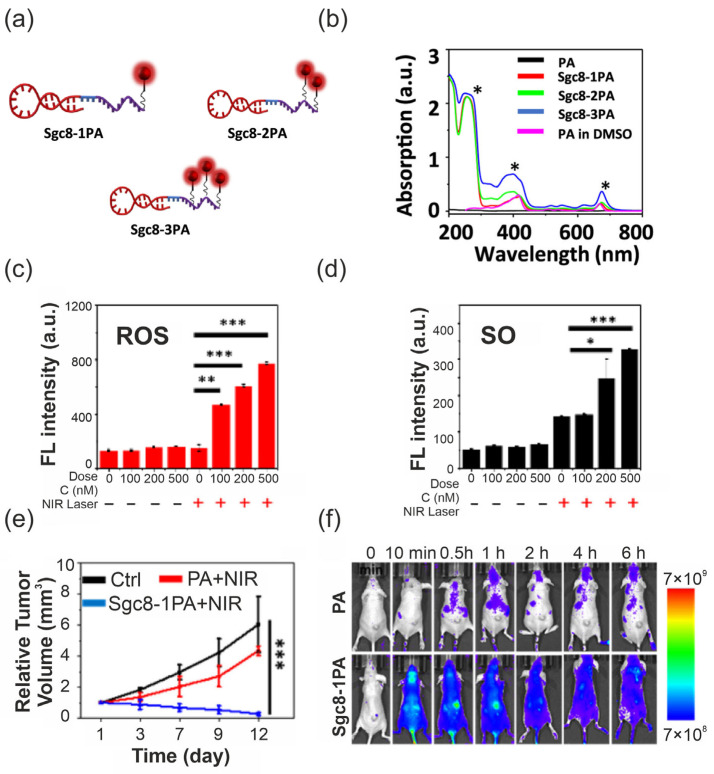
Overview of the Sgc8 aptamer-pyropheophorbide a platform for targeted PDT. (**a**) Schematic representation of the molecular design of the aptamer-PA conjugate. (**b**) Electronic absorption spectra confirming the conjugation. (**c**,**d**) Evaluation of intracellular oxidative stress: analysis of (**c**) ROS and (**d**) ^1^O_2_ generation in HCT 116 cells treated with 500 nM Sgc8-1PA under NIR irradiation, assessed via confocal microscopy and flow cytometry. (**e**) In vivo therapeutic efficacy demonstrated by tumour growth inhibition curves over a 12-day treatment period. (**f**) Time-dependent in vivo fluorescence imaging tracking the biodistribution and tumour accumulation of Sgc8-1PA compared to free PA in HCT 116 tumour-bearing mice. Data are the mean ± SD of three independent experiments (two-way variance analysis (ANOVA) with Bonferroni *t*-test: * *p* < 0.05, ** *p* < 0.01, *** *p* < 0.001). Adapted from [129] and modified.

The therapeutic potential of this self-delivering system was validated in human colorectal carcinoma tumour-bearing mice. The study reported that the conjugate facilitated rapid tumour accumulation and, following irradiation with a 660 nm laser, yielded a tumour inhibition rate more than 20-fold higher than that of the free photosensitizer, with no observed systemic toxicity [129]. The comprehensive characterization of this conjugate, including its molecular design, spectroscopic properties, intracellular ROS detection, and in vivo therapeutic efficacy, is summarized in Figure 8.

In the context of specific oncological targets, the G-rich oligonucleotide aptamer AS1411 is among the most extensively studied. AS1411 forms a stable G-quadruplex structure and specifically targets nucleolin, a protein overexpressed on the surface of rapidly proliferating cancer cells. Studies have indicated that conjugating photosensitizers, such as chlorin e6, to AS1411 significantly enhances cellular uptake via receptor-mediated endocytosis [141]. Expanding the structural diversity of such conjugates, recent investigations have also reported the synthesis of novel indium(III) phthalocyanines covalently linked to AS1411. The incorporation of the central indium ion utilizes the heavy atom effect to enhance singlet oxygen quantum yields, while the aptamer ensures selective internalization in nucleolin-overexpressing breast cancer cells [143]. Similarly, the MUC1 aptamer was employed to form conjugates with Ce6 for the treatment of epithelial cancers; these constructs are often engineered as ‘switchable’ probes, designed to release or activate their payload only following specific interaction with the receptor [142]. Beyond direct targeting, aptamer conjugates are increasingly being designed to modulate TME, particularly to overcome hypoxia, which severely limits Type II PDT mechanisms. For instance, a multifunctional conjugate was developed for bladder cancer that co-delivers Ce6 and the enzyme catalase using a tumour-specific aptamer. In this system, the catalase component decomposes endogenous hydrogen peroxide (H_2_O_2_) within the TME to generate oxygen in situ. This oxygen self-supply strategy successfully fuels the photodynamic reaction, resulting in significantly enhanced tumour growth inhibition in vivo compared to conventional treatments [144]. These examples illustrate the capacity of aptamers to serve not merely as passive targeting vectors, but as functional scaffolds for advanced, environment-responsive phototherapy. By integrating target recognition with oxygen-modulating enzymes or other TME-responsive elements, aptamer–PS conjugates exemplify how nucleic acid scaffolds can be used to program both the spatial and biochemical dimensions of photodynamic action.

### 4.3. Vitamins and Coenzymes

Vitamins and coenzymes have emerged as a distinct class of low-molecular-weight ligands for the targeted delivery of PSs. Owing to their essential roles in cellular metabolism, these biomolecules are taken up by cells through highly specific receptors or transport systems, many of which are upregulated in rapidly proliferating or metabolically active cells. This characteristic has been widely exploited in the design of PS conjugates aimed at achieving receptor- or transporter-mediated internalization. From a molecular design perspective, vitamins and coenzymes offer several advantages as delivery vectors. They are generally non-immunogenic, chemically well-defined, and sufficiently small to be conjugated to photosensitizers without severely altering their photophysical properties. Covalent attachment can be achieved either directly or via suitable linkers, allowing flexibility in controlling solubility, intracellular trafficking, and pharmacokinetic behaviour of the resulting conjugates. As a result, a broad range of vitamin- and coenzyme-based PS conjugates has been reported, encompassing porphyrins, chlorins, bacteriochlorins, and phthalocyanines. Among the most widely explored systems are conjugates incorporating small-molecule vitamins or vitamin-derived cofactors that exploit endogenous uptake pathways, such as receptor-mediated endocytosis or active transport mechanisms. In many cases, this strategy leads to enhanced cellular accumulation of the PS and improved photodynamic performance compared to non-conjugated analogues, while maintaining favourable biocompatibility profiles [145].

Folic acid and its derivatives are commonly employed in PS conjugates due to the overexpression of folate receptors in many cancer cells and inflammatory tissues. Conjugation with folate facilitates receptor-mediated internalization, enhancing selective accumulation of photosensitizers in target cells. The proper design of the folate ligand and the attached PSr enables modulation of pharmacokinetic properties, solubility, and intracellular distribution, ultimately enhancing the therapeutic performance of photodynamic treatments [146]. Pyropheophorbide a conjugated with folic acid increased the accumulation of the PS inside the tumours, enhancing photodynamic efficacy in the human mouth epidermal carcinoma model [147]. The enhanced toxicity against folate receptor (FR) overexpressing cells was assessed using folate-conjugated platinum porphyrin complex [148].

Vitamin B12 derivatives enable the construction of photolabile conjugates, in which the photoactive PS can be released upon light excitation. Studies on B12 conjugates demonstrated that structural modifications of cobalamin allow selective uptake by cancer cells while providing controlled release of the PS at the target site, potentially enhancing photodynamic efficacy and minimizing off-target toxicity [149]. BODIPY dye attached to the vitamin B12 was designed as a fluorescent probe for direct visualization of cellular uptake, localization, and light-triggered release. Second, it functions as a light-absorbing antenna that enables excitation at longer wavelengths than native cobalamin absorption, facilitating photolysis under biologically relevant conditions. BODIPY–Cbl conjugate was shown to be stable in the dark, with no spontaneous release of the BODIPY ligand. Upon illumination at wavelengths absorbed by the BODIPY chromophore (green to red light, depending on substitution), energy transfer from the excited BODIPY to the cobalamin core induces homolytic cleavage of the Co–C bond, releasing free BODIPY. In HeLa cells, fluorescence microscopy revealed a clear change in fluorescence distribution after irradiation, consistent with intracellular photorelease of the dye. This provided direct evidence that light activation can occur inside living cells [150].

Riboflavin and its derivatives also exhibit intrinsic photosensitizing properties, particularly in the context of skin cancer treatment. Both natural and synthetic riboflavin derivatives can generate ROS upon light exposure, leading to selective cytotoxicity in tumour cells. Their small size and biocompatibility facilitate chemical modification without significantly altering photophysical characteristics [151].

Biotin, as a highly specific vitamin ligand, has been conjugated to chlorin e6. Such conjugates demonstrate enhanced internalization in cancer cells via biotin receptors, improving photodynamic efficacy in vitro. Biotin-PS conjugates also allow better control over intracellular distribution and reduce undesired effects in healthy cells [152]. Building on this targeting strategy, recent work has shown that biotinylation can exert an additional photochemical effect beyond receptor-mediated uptake. Specifically, covalent attachment of biotin to PSs was found to significantly enhance the generation of superoxide anion radicals (O_2_^•−^) under irradiation, while maintaining the ability to produce singlet oxygen via Type II mechanism. This dual ROS-generating profile is attributed not to a direct redox activity of biotin itself, but rather to biotin-induced changes in the local microenvironment and photophysical balance of the PS, which favor photoinduced electron transfer processes alongside conventional energy-transfer mechanisms. As a result, biotinylated photosensitizers can achieve effective phototoxicity even under hypoxic conditions typical of solid tumours, combining tumour targeting with hypoxia-tolerant PDT performance [153].

In addition to traditional PSs design strategies, endogenous coenzymes have attracted growing interest in PDT due to their central role in cellular redox homeostasis and electron transfer processes. Coenzymes such as NADH are key participants in intracellular redox balance and serve as electron donors in numerous oxidoreductase-mediated reactions. Recent work has highlighted strategies that harness photocatalytic NADH oxidation to perturb mitochondrial electron transport and enhance ROS generation, including superoxide ion production. In this case, excited PS extract electrons from NADH, forming radical intermediates that can transfer electrons to oxygen, thereby generating reactive oxygen species through charge-transfer pathways and Type I mechanisms [94].

### 4.4. Amino Acids

Amino acid modification represents a simple yet powerful strategy to fine-tune the physicochemical properties of photosensitizers. By conjugating amino acids via amide or ester bonds, one can systematically modulate lipophilicity, charge distribution, and membrane affinity, thereby improving dispersibility and cellular permeability without substantially compromising the intrinsic photophysical behaviour of the chromophore [154].

Furthermore, the choice of amino acid can impart additional biological functionality. For example, arginine or lysine residues can enhance electrostatic interactions with negatively charged cell membranes, while aromatic amino acids such as tryptophan or phenylalanine can promote π–π stacking with cellular biomolecules, potentially stabilizing the PS at target sites. In this way, amino acid conjugation can influence both the biodistribution and the photodynamic performance of the PS. Moreover, amino acid substitution frequently modulates the aggregation behaviour of the chromophore, either suppressing non-productive π–π stacking or promoting controlled self-assembly into nano-objects with distinct cellular uptake and clearance profiles [155,156,157].

Among currently available photosensitizes, chlorin e6 conjugated with specific amino acids has been studied extensively. One of the most clinically relevant conjugates is talaporfin sodium (mono-L-aspartyl chlorin e6; also known as NPe6). This molecule achieves an optimal balance between hydrophilicity and photodynamic efficiency, enabling good cellular uptake and effective singlet oxygen generation, while maintaining low dark toxicity. The safety and efficacy of talaporfin have been validated in both in vitro and in vivo models, leading to its clinical use in Japan for PDT of lung and oesophageal cancers [158].

A particularly innovative strategy was previously highlighted, involving the synthesis of a silicon(IV) phthalocyanine in which arginine was axially coordinated to the central silicon atom rather than attached to the macrocyclic periphery. This axial coordination preserves the π-conjugated system of the phthalocyanine, while drastically improving water solubility, cellular internalization, and light-triggered cytotoxicity. In vitro studies conducted on HeLa, MCF-7, and HuH-7 cell lines confirmed strong photodynamic activity and pro-apoptotic effects. This architecture therefore represents a refined method of improving bioavailability without perturbing the core photophysical characteristics of the PS [159].

Beyond small-molecule conjugates, amino acid modification has also been exploited in macromolecular systems. For instance, in a recent study, chlorin e6 was conjugated to a poly-L-lysine/polyphenylalanine polypeptide to yield self-assembling micelles that co-deliver chemotherapeutic cargo together with the PS. These polypeptide-Ce6 micelles display enhanced cellular uptake in acidic tumour-like environments, and upon irradiation, synergistically induce cancer cell death through combined photodynamic and chemotherapeutic mechanisms [160].

**Figure 9:**
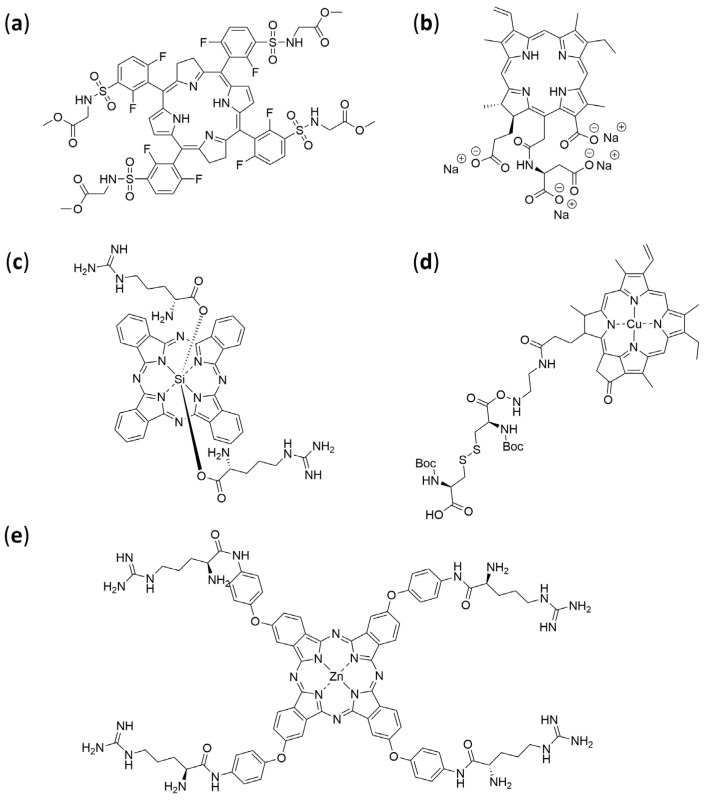
Representative structures of amino acid–photosensitizer conjugates (**a**) Bacteriochlorin–glycine conjugate; (**b**) Chlorin e6–aspartic acid conjugate; (**c**) Si(IV) phthalocyanine–arginine conjugate; (**d**) Cu(II) Pyropheophorbide-a–cysteine conjugate; (**e**) Zn(II) phthalocyanine–arginine conjugate.

Collectively, the literature supports that amino acid conjugation is a versatile and effective tool for improving PS performance: by carefully selecting the type of amino acid, the site of conjugation, and the chemical linkage, one can modulate solubility, targeting, and phototoxicity in a rational and tenable manner. These strategies remain highly relevant for the design of next-generation photosensitizers with improved therapeutic indices and better clinical translatability. Table 5 and Figure 9 summarize notable amino acid-photosensitizer conjugates reported to date, together with the experimental models employed to evaluate their photophysical properties, biological activity, and therapeutic potential.

**Figure 10:**
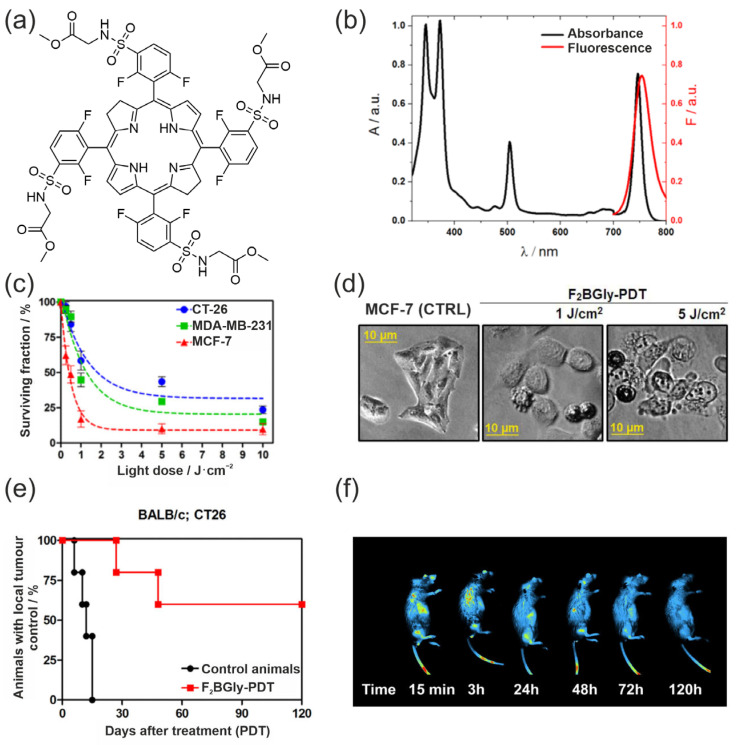
(**a**) Structure of the glycine-conjugated bacteriochlorin (F_2_BGly). (**b**) Absorption and fluorescence spectra of F_2_BGly in ethanol. (**c**) In vitro phototoxicity of F_2_BGly against CT-26, MDA-MB-231, and MCF-7 cancer cells. (**d**) Morphological alterations in MCF-7 cells were observed after F_2_BGly-PDT using light doses of 1 J/cm^2^ and 5 J/cm^2^ compared to controls. (**e**) Survival rates of CT26 tumour-bearing mice treated with F_2_BGly-PDT. (**f**) Time-lapse in vivo fluorescence imaging of F_2_BGly accumulation in CT26 tumours in BALB/c mice post-i.v. injection (λ_ex_ = 680 nm, λ_em_ = 750 nm). Adapted from [54] and modified.

Amino acid conjugation also plays a role in the design of next-generation bacteriochlorin photosensitizers, exemplified by F_2_BGly, a molecule developed in our laboratory by introducing four glycine residues onto a fluorinated phenyl groups within bacteriochlorin core. This modification resulted in a highly stable NIR-absorbing chromophore with markedly improved amphiphilicity and preserved monomeric character in biological media, as reflected by the well-defined absorption and fluorescence spectra shown in Figure 10a,b.

The glycine residues exert a dual functional effect, enhancing cellular uptake and retention while simultaneously stabilizing the photophysically active form of the molecule. Consistent with these structural features, F_2_BGly displays potent light-dependent cytotoxicity across several cancer cell lines (Figure 10c), accompanied by characteristic morphological signatures of PDT-induced damage (Figure 10d). In vivo evaluation in the CT26 murine model further demonstrated that F_2_BGly achieves efficient tumour accumulation, enables real-time fluorescence imaging, and mediates robust tumour control following irradiation (Figure 10e,f). Mechanistic analyses confirmed that F_2_BGly can engage both Type I and Type II photochemical pathways, supporting combined tumour-cell and vascular targeting and offering resilience under hypoxic conditions typical for solid tumours.

Taken together, properties such as rational modification, favourable supramolecular organization, efficient ROS generation, and strong therapeutic performance identify F_2_BGly as a representative example of how tailored biomolecular conjugation can translate into advanced, clinically relevant PDT agents [161].

### 4.5. Peptides

**Figure 11:**
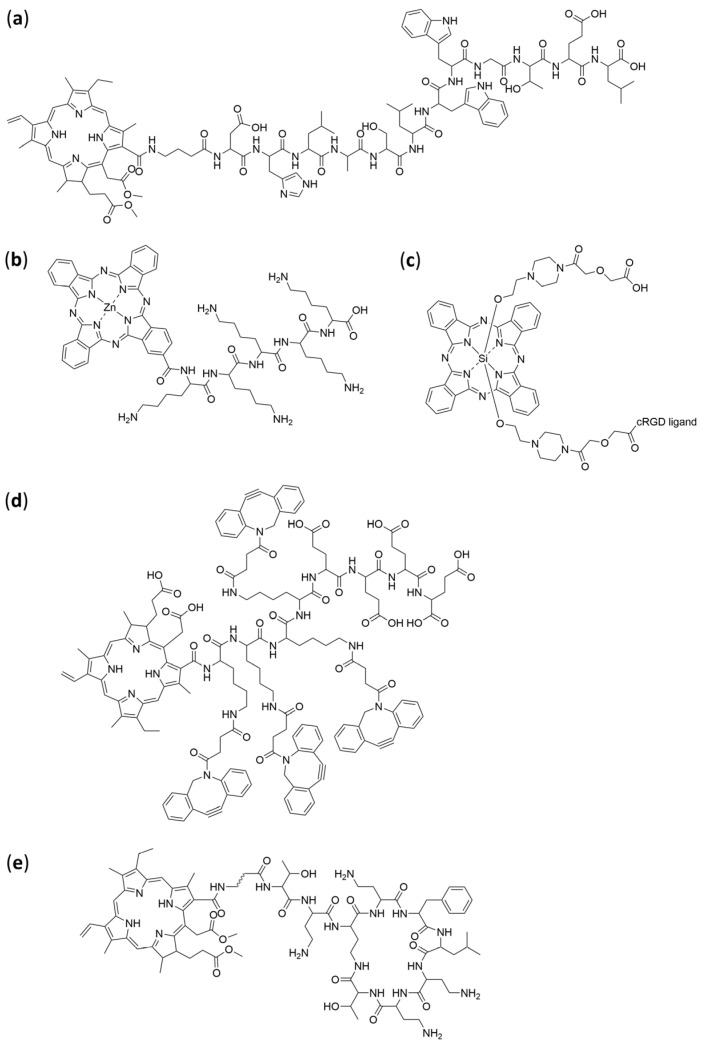
Representative structures of peptide–photosensitizer conjugates (**a**) Chlorin e6–GPC3-targeting peptide conjugate; (**b**) Zn(II) phthalocyanine-(Lys)_5_ conjugate; (**c**) Si(IV) phthalocyanine–cRGD conjugate; (**d**) Chlorin e6–P-DBOC conjugate; (**e**) Chlorin e6–Cyclic polymyxin-derived peptide (PMBN) conjugate.

Photosensitizer-peptide conjugates remain a cutting-edge strategy in PDT, combining the phototoxic efficacy of classic PS with the high specificity and favourable pharmacokinetics conferred by peptides. These conjugates enable targeted delivery, enhanced tumour accumulation, improved cellular uptake, and better biocompatibility (Table 6, Figure 11).

Peptides used in PS conjugates often function as targeting ligands (e.g., receptor-binding peptides) or as cell-penetrating peptides (CPPs). The rationale is to exploit receptor-mediated uptake or facilitate membrane translocation to enhance PS internalization into tumour cells. According to a recent review, peptide-PS bioconjugation addresses major limitations of conventional PDT, such as poor accumulation of PS in tumours and off-target toxicity [55,99]. Peptide conjugation can also improve the physicochemical properties of PS, for example, by increasing hydrophilicity and aqueous dispersibility, modulating aggregation behaviour, and reducing nonspecific plasma protein binding. Importantly, recent studies leverage biorthogonal “click” chemistry and metabolic glycoengineering to covalently anchor PS-peptide conjugates to the cell membrane, thereby prolonging tumour retention [165].

**Table 6:** Examples of peptide–photosensitizer conjugates evaluated in cancer and bacterial experimental models.

Peptide	Photosensitizer	Model	Reference
GPC3-targeting peptide	Chlorin e6	Human hepatocellular carcinoma	[166]
(Lys)_5_	Zn(II) phthalocyanine	Bacterial Skin Infections	[167]
		Human hepatocellular carcinoma	
cRGD	Si(IV) phthalocyanine	Human glioblastoma	[168]
		Human prostate carcinoma	
		Human prostate adenocarcinoma	
		Human epidermoid carcinoma	
P-DBOC	Chlorin e6	Human cervical cancer	[165]
Cyclic polymyxin-derived peptide (PMBN)	Chlorin e6	Gram-negative bacterial infections	[169]

Glypican-3-targeting peptide–chlorin e6 conjugates have also been developed for hepatocellular carcinoma (HCC). In this approach, a GPC3-binding peptide is conjugated to chlorin e6 to exploit the overexpression of glypican-3, a tumour-associated proteoglycan characteristic of HCC cells. This conjugate shows high uptake in GPC3-positive cells and, in a HepG2 xenograft model, achieved complete tumour eradication upon light activation while sparing healthy tissues [166].

**Figure 12:**
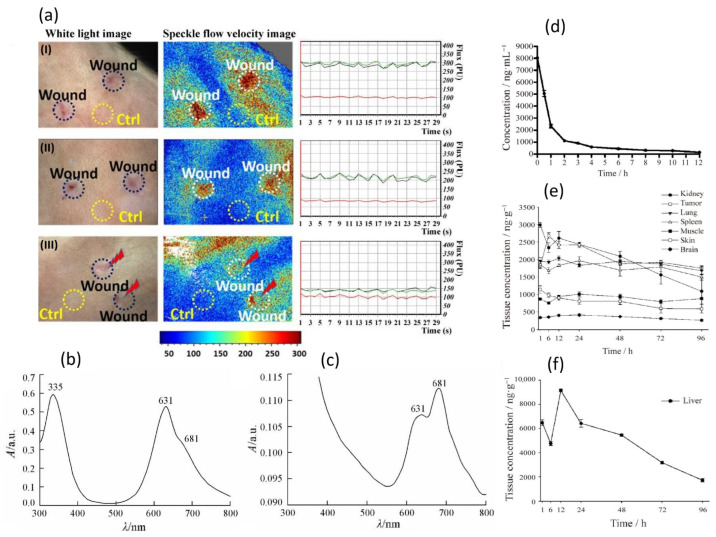
(**a**) Representative speckle images illustrate the changes in wound perfusion following administration of ZnPc-S4 (I) and ZnPc(Lys)_5_, shown both without irradiation (II) and after light exposure at 680 nm (15 J/cm^2^) (III). Electronic absorption spectra of ZnPc(Lys)_5_ recorded in PBS (**b**,**c**) in the presence of K562 cells. (**d**–**f**) Biodistribution studies performed in S180 tumour-bearing mice (*n* = 5) demonstrate the in vivo accumulation profile of ZnPc(Lys)_5_ across major organs, providing insight into systemic distribution and tumour selectivity. Adapted from [167] and modified.

Phthalocyanine-peptide conjugates address two major limitations of phthalocyanines: poor water solubility and limited tumour selectivity. Conjugating zinc(II) phthalocyanine with a hydrophilic peptide, such as pentalysine, has been shown to markedly enhance its solubility and tumour targeting. In a 2024 study, the resulting conjugate ZnPc(Lys)_5_ demonstrated strong phototoxicity and favourable biocompatibility, broadening its potential applications not only in cancer therapy but also in antibacterial PDT (Figure 12) [167].

Cyclic peptide-chlorin conjugates have also been explored for aPDT. Beyond oncology, peptide-photosensitizer conjugates show considerable promise in antimicrobial applications. A cyclic nonapeptide derived from polymylxin B, which selectively binds lipopolysaccharide on Gram-negative bacteria, has been conjugated to chlorin e6. These conjugates display enhanced ROS generation, improved aqueous dispersibility, and selective bactericidal activity upon 660 nm irradiation [169].

A classic example is the silicon phthalocyanine-cRGD conjugate, in which the cRGD motif targets the αvβ3 integrin overexpressed on tumour cells. In preclinical models, this conjugate achieved efficient tumour eradication with minimal regrowth, underscoring its strong therapeutic potential [168].

### 4.6. Proteins, Antibodies, and Affibodies

Protein-based conjugates, particularly antibody–photosensitizer systems, have emerged as one of the most clinically advanced PDT strategies [89]. These conjugates combine the high target specificity of monoclonal antibodies with the phototoxic potential of photosensitizers, forming the foundation of photoimmunotherapy (PIT) [170,171,172,173,174].

**Figure 13:**
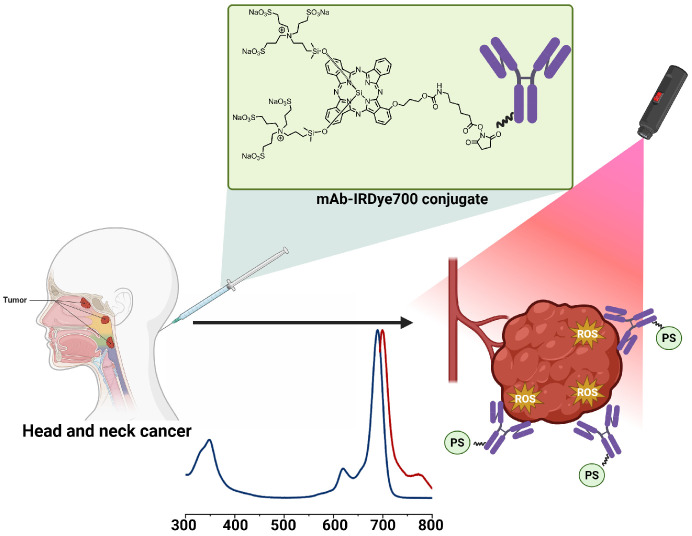
Mechanism of action of the cetuximab–IR700 (ASP-1929) conjugate in targeted phototherapy for head and neck cancer. Adapted from [175] and created in BioRender. Warszyńska, M. (2025) https://BioRender.com/61fpsl1 (accessed on 18 December 2025).

A prominent example is IR700 (IRdye700DX), a near-infrared photosensitizer covalently linked to various antibodies (Figure 13). IR700 conjugated with cetuximab, known as ASP-1929, represents one of the most clinically advanced phototherapeutic agents to date. Currently, a Phase 3 clinical trial is actively recruiting participants to further evaluate the efficacy of the cetuximab-IR700 conjugate (ASP-1929) in combination with anti-PD-1 therapy for patients with recurrent or metastatic head and neck squamous cell carcinoma (NCT06699212) [175]. Upon irradiation, these conjugates induce rapid necrotic cell death selectively in receptor-expressing tumour cells while sparing healthy tissue.

However, the understanding of the mechanism driving this cytotoxicity has evolved significantly beyond classical PDT. Recent experimental work employing trastuzumab-IR700 conjugates against HER2-positive breast cancer cells proposed a novel, intriguing mode of action. It was demonstrated that the therapeutic effect is not primarily driven by ROS generation. Instead, NIR irradiation induces the photochemical cleavage of the hydrophilic axial ligands from the silicon-phthalocyanine core of the IR700 molecule. This reaction triggers a rapid transition of the conjugate from a hydrophilic to a highly hydrophobic state, leading to precipitation and aggregation on the cell surface. This massive aggregation physically disrupts cell membrane integrity, causing water influx and immediate necrosis. This membrane-permeabilizing mechanism functions effectively even in low-oxygen environments, distinguishing IR700 conjugates from conventional Type II PSs [176].

Beyond antibodies, smaller protein scaffolds known as affibodies (~7 kDa) have gained attention due to their high binding affinity, excellent stability, and superior tumour penetration compared with full-length antibodies (Table 7). IR700–affibody conjugates targeting HER2 or EGFR receptors have shown promising preclinical outcomes, suggesting their potential for next-generation targeted-PDT.

Recent studies with HER2-targeted affibody–PS conjugates illustrate these general principles in detail. Among the various targeting ligands, affibodies, small, engineered protein mimetics, have gained considerable attention due to their high binding affinity, stability, and rapid tumour accumulation. The conjugation of this affibody to the IRDye700 photosensitizer enabled near-infrared photoimmunotherapy (NIR-PIT) with selective cytotoxicity toward HER2-positive breast cancer cells [178]. Moreover, conjugation of the same affibody to the pyropheophorbide-a photosensitizer yielded potent photodynamic effects both in vitro and in vivo, highlighting the advantages of small, high-affinity ligands over conventional antibodies, including enhanced tumour penetration, rapid clearance, and improved specificity. Together, these findings establish classical HER2-targeting affibodies as versatile platforms for precision phototherapeutics [177].

Recent comprehensive reviews of EGFR-targeted PDT have highlighted that EGFR is one of the most frequently overexpressed receptors in tumours such as glioblastoma, lung, colorectal, and head and neck cancers. Conjugating PSs with EGFR-recognizing ligands, including monoclonal antibodies, antibody fragments, affibodies, peptides, and small-molecule inhibitors, greatly enhances the selective delivery of PSs to cancer cells. However, regarding glioblastoma treatment, the blood–brain barrier (BBB) remains a critical physiological hurdle that severely limits the accumulation of systemically administered agents. While full-length antibodies (~150 kDa) struggle to cross the BBB, smaller scaffolds like affibodies (~7 kDa) offer significantly better tissue penetration and diffusion properties. Although crossing the intact BBB remains challenging, the compromised integrity of the BBB in high-grade gliomas, combined with the small size of affibody conjugates, presents a more viable strategy for delivering sufficient PS loads to intracranial tumours compared to larger antibody-based vehicles. Unlike classical EGFR inhibitors, targeted PDT uses the receptor solely as a docking site, enabling effective killing of cells that are resistant to targeted therapies. The review also highlights challenges, such as the immunogenicity of large bioligands and limited light penetration, while discussing modern solutions, including NIR-activatable photosensitizers, PEGylation, and PTT (photothermal therapy) combination strategies. Overall, EGFR-targeted PDT emerges as a highly selective and promising therapeutic platform with strong translational potential [179].

**Figure 14:**
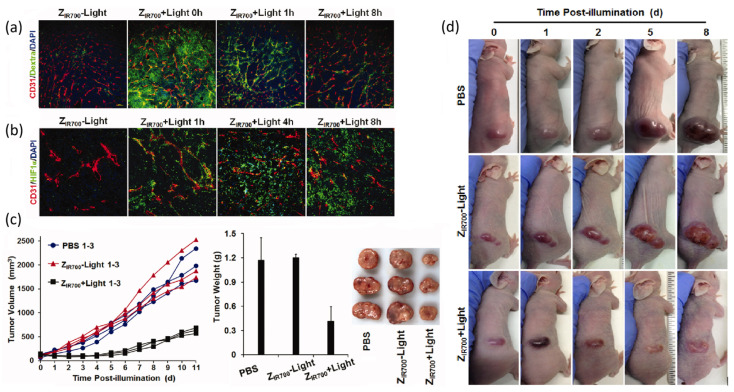
Z_IR700_-mediated PDT in LS174T tumour-bearing mice. Mice with the LS174T tumour were injected with Z_IR700_ and either illuminated (Z_IR700_ + Light) or kept non-illuminated (Z_IR700_ − Light). (**a**) Tumour blood vessel permeability assessed by FITC-dextran leakage and CD31 staining at 0–8 h post-illumination. (**b**) HIF-1α expression in tumour tissues at 1–8 h post-illumination. (**c**) Tumour growth suppression after PDT. Mice (*n* = 3) received Z_IR700_ and illumination on days 0 and 3. (**d**) Summary of Z_IR700_-mediated PDT efficacy in suppressing LS174T tumour growth. Adapted from [182] and modified.

A vascular-targeted photodynamic therapy (V-PDT) directed at PDGFRβ employs a high-affinity affibody linked to the IR700. Unlike cellular-targeted approaches that aim to destroy malignant cells directly, V-PDT focuses on the selective disruption of tumour vasculature to deprive the neoplasm of oxygen and nutrients (Figure 14). The affibody specifically binds PDGFRβ, a receptor highly expressed on tumour-associated pericytes. After intravenous administration in mice bearing LS174T colorectal tumours, the affibody-IR700 conjugate accumulated selectively in the tumour vasculature but not in tumour cells themselves. Upon NIR irradiation, the conjugate generated large amounts of ROS, causing rapid destruction of tumour blood vessels, including increased permeability, vessel collapse, and thrombosis. This led to pronounced tumour hypoxia and significant inhibition of tumour growth. The therapeutic effect was further enhanced when combined with TNF-related apoptosis-inducing ligand (TRAIL), showing that vascular disruption improved selectivity and anticancer activity. This study demonstrates that PDGFRβ-specific affibody targeting enables highly selective delivery of PSs to tumour vasculature, resulting in effective and minimally invasive V-PDT for colorectal cancer, with potential applicability to other tumour types exhibiting PDGFRβ overexpression [182].

In recent years, antibody-photosensitizer conjugates have emerged as one of the most rapidly advancing approaches in PDT resistance mechanisms (Table 8). A notable example is the HER2-targeted IRDye700DX conjugate, which exhibits strong specificity toward HER2-overexpressing cancer cells and can overcome resistance to standard anti-HER2 therapy while simultaneously serving as an imaging agent. Upon light activation, the conjugate induces potent phototoxicity driven by singlet oxygen generation, resulting in pronounced tumour growth inhibition and offering a promising theranostic platform [187,188].

Another important innovation is the strategy employing an anti-EGFR-chlorin e6 conjugate whose therapeutic effect remains robust regardless of KRAS mutational status-one of the major obstacles in treating colorectal cancer. The combination of PDT with receptor-targeted delivery not only induces extensive cell death upon irradiation but also suppresses key proliferative pathways and stimulates immune system activation, yielding a strong synergistic response and substantial tumour reduction in vivo [109].

Significant advances are also represented by nanobody-based platforms, including a self-reporting conjugate in which the PS is released upon ROS generation, restoring fluorescence and enabling real-time visualization of photodynamic performance. This mechanism ensures prolonged intratumoral retention of the PS despite rapid systemic clearance of the nanobody itself, allowing precise and sustained treatment of large tumour volumes while maintaining excellent tolerability [112].

Another approach utilizes gold nanoparticle carriers functionalized with a melanoma-specific antibody and a phthalocyanine photosensitizer. This multifunctional nanoconstruct provides high stability and enhanced tumour accumulation of the PS, while the gold nanoparticles further promote ROS generation due to their photophysical properties. Studies performed in both 2D monolayers and 3D tumour spheroids demonstrate markedly increased phototoxicity compared to the bare PS, highlighting the potential of nanoscale carriers to improve PDT efficacy in structurally complex tumours [192,195].

A distinct direction is represented by near-infrared photoimmunotherapy targeting PD-L1, allowing the combination of selective destruction with modulation of immune checkpoint pathways. In an ovarian cancer model, the PD-L1-IR700 conjugate leads to strong suppression of tumour growth following irradiation while simultaneously reshaping the tumour microenvironment by reducing immunosuppressive markers. This suggests added immunomodulatory benefits and aligns with the growing trend toward designing phototherapies with dual mechanisms-direct cytotoxicity coupled with immune system reprogramming [193].

**Figure 15:**
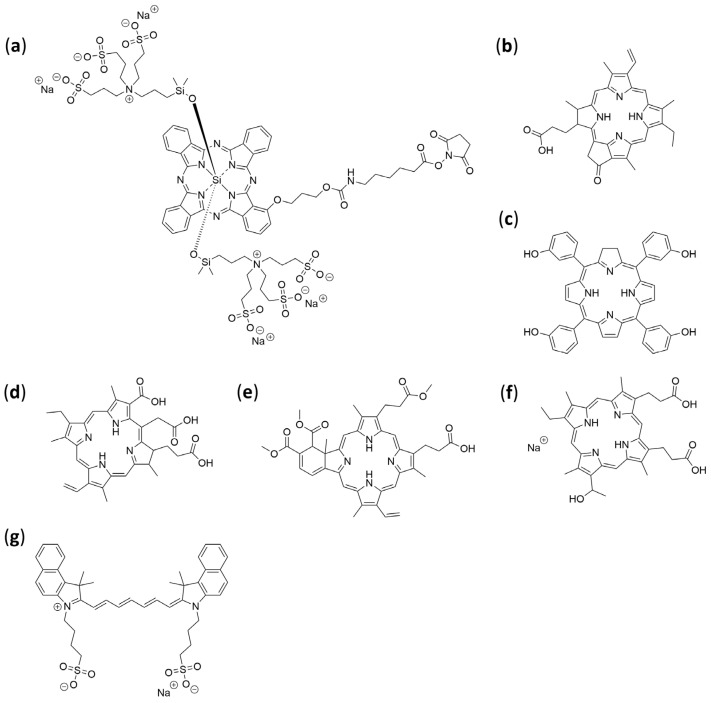
Representative structures of PSs used in conjugates with proteins, antibodies, and affibodies (**a**) IRDye700DX; (**b**) Pyropheophorbide-a; (**c**) Temoporfin; (**d**) Chlorin e6; (**e**) Verteporfin; (**f**) Photofrin; (**g**) Indocyanine green.

Photosensitizer–protein conjugates and protein-assisted nanoassemblies have emerged as powerful strategies in PDT, as they improve the physicochemical properties of PSs while imparting biological functionality such as enhanced solubility, prolonged circulation, and selective tumour targeting (Table 9, Figure 15). The conjugation of cyclometalated Ir(III) complexes to human serum albumin (HSA) enhances the solubility and stability of the PS in biological environments, facilitating its intracellular uptake and enabling efficient generation of ROS upon light irradiation, which leads to oncosis-mediated cell death rather than classical apoptosis. Similarly, the encapsulation of IR820 in liposomes modified with transferrin allows targeted delivery to cancer cells overexpressing transferrin receptors, resulting in increased accumulation of the PS within the tumour tissue and enhanced induction of apoptosis upon near-infrared light exposure, while minimizing toxicity in non-target tissues [196]. In both cases, the formation of defined nano-assemblies with serum proteins or liposomal carriers underscores how protein-based conjugation and encapsulation strategies intrinsically couple targeting with control over supramolecular organization. In a complementary approach, bioluminescence-mediated PDT utilizes luciferase enzymes as an internal light source to activate PSs such as Chlorin e6 or Rose Bengal without external illumination, enabling the treatment of deep-seated tumours that are otherwise inaccessible to traditional PDT regimes. This strategy relies on efficient energy transfer from the bioluminescent donor to the PS and demonstrates high selectivity and potential safety in preclinical models [197].

### 4.7. Self-Assembling Aggregates and Supramolecular Photosensitizer Systems

While the conjugation of photosensitizers with biomolecules, such as aptamers or antibodies, discussed in the preceding sections, offers a potent strategy for targeted delivery and enhanced selectivity, the fundamental physicochemical behaviour of the PS core itself remains a critical determinant of therapeutic efficacy. Beyond the biological interactions facilitated by targeting ligands, the intrinsic tendency of these planar aromatic macrocycles to self-assemble via strong π–π interactions in physiological environments presents a complex variable that dictates the final photodynamic outcome. Such aggregation has often been regarded merely as a physicochemical impediment necessitating mitigation through chemical modification or bioconjugation. However, emerging evidence indicates that these supramolecular assemblies’ function not as inert reservoirs of deactivated PSs, but rather as dynamic, environment-responsive systems that can be engineered to facilitate advanced therapeutic modalities.

The impact of self-assembly on photophysical properties is profound. Intermolecular coupling within an aggregate alters the electronic landscape of the molecule, typically resulting in the splitting of excited state energy levels. In the most common scenario, H-aggregates, the transition to the lowest excited state becomes forbidden, favouring rapid non-radiative decay via thermal dissipation. This process drastically reduces the quantum yield of the triplet excited state, thereby suppressing the generation of ROS, effectively rendering the PS inactive in standard assays. Conversely, specific molecular arrangements (J-aggregates) induce a bathochromic shift in absorption and may retain the ability to generate ROS, challenging the paradigm that all aggregation is inherently unfavourable to PDT [201,202].

To control these phenomena, structural engineering of the porphyrin or phthalocyanine macrocycles has proven essential. Recent research indicates that the specific arrangement of substituents plays a pivotal role in dictating the aggregation pathway. For instance, placing cationic substituents at non-peripheral (α) positions introduces significant steric hindrance that restricts conformational freedom, effectively suppressing aggregation in PBS and preserving the monomeric, photoactive state. In stark contrast, analogues with peripheral (β) substitution tend to form highly stable, photo-inactive H-aggregates. Furthermore, the introduction of amphiphilic features, such as octaethylene glycol chains, has been shown to facilitate the formation of stable nanostructures (e.g., NanoNMO) that resist uncontrolled size expansion in the presence of serum proteins, thereby enhancing physiological stability for systemic delivery [202,203].

However, a critical factor often overlooked in standard spectroscopic characterization is the dynamic response of these aggregates to the complex biological microenvironment. Recent studies have demonstrated that the photo-inactivity observed in simple buffer solutions does not necessarily predict therapeutic failure in vitro. It has been observed that highly aggregated, non-fluorescent cationic Pcs can exhibit potent nanomolar phototoxicity (IC_50_ values ranging from 27 to 358 nM) against cancer cells, indistinguishable from their monomeric counterparts. This apparent paradox is explained by the intracellular disassembly of aggregates into photoactive monomers, a process likely driven by specific interactions with lipid membranes and intracellular components. This activation pathway, predicated on aggregate dissociation, is critically regulated by serum proteins, most notably albumin (BSA), which functions as an endogenous facilitator of monomerization within the systemic circulation [204]. Upon binding to BSA, aggregated Pcs dissociate to form protein-bound monomers, leading to a recovery of fluorescence and ROS generation capabilities. The affinity for albumin varies significantly with molecular structure; for example, the octa-cationic 8βZnPc exhibits an exceptionally high binding constant (12.6 × 10^6^ M^−1^), resulting in a more than 4-fold enhancement of its photodynamic activity in the presence of fetal bovine serum (FBS) compared to serum-free conditions [202].

Beyond the paradigm of disassembly, recent breakthroughs have introduced the concept of utilizing the aggregate state itself as a functional therapeutic agent by fundamentally shifting the photochemical mechanism. While monomeric Pcs typically rely on Type II (energy transfer) pathway, specific nanostructured Pc aggregates can function as semiconductor-like photocatalysts. Through a process known as photoinduced symmetry-breaking charge separation (SBCS), these aggregates facilitate a self-substrate mechanism wherein neighbouring Pc molecules function simultaneously as electron donors and acceptors to generate radical ion pairs (Pc^•+^ and Pc^•−^). This electronic reconfiguration enables superoxide ions (O_2_^•−^) generation via a Type I mechanism. Importantly, this feature is clinically relevant, as it enables effective tumour treatment even under hypoxic conditions, where traditional Type II photosensitizers are less effective. Such semiconductor-like nanodots have demonstrated not only superior tumour inhibition but also the ability to trigger ICD and synergize with immune checkpoint inhibitors (anti-PD-1) to suppress metastasis. Thus, the aggregation of PSs is evolving from a simple formulation hurdle into a sophisticated tool for designing smart, environment-responsive, and mechanism-tunable nanomedicines [205]. These principles are equally relevant for biomolecule–photosensitizer conjugates, where the choice of targeting ligand, linker, and substitution pattern can be used not only to direct receptor binding but also to programme aggregation, disassembly, and a controlled switch between Type I and Type II photochemical mechanisms in vivo.

**Figure 16:**
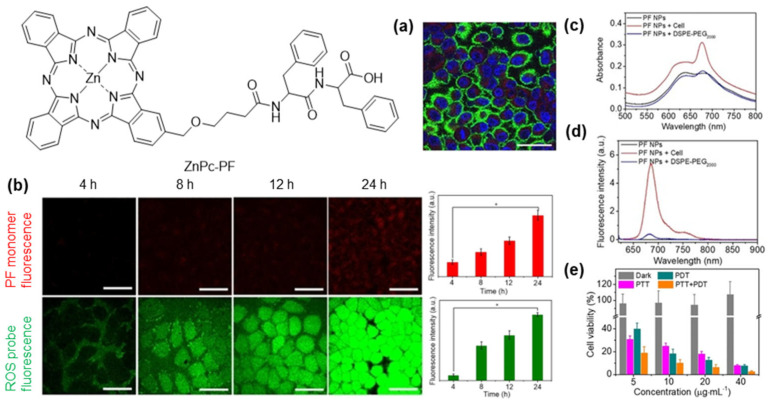
Structure of peptide–phthalocyanine conjugate (**a**) Image of MCF-7 cells incubated with PF NPs for 24 h. (**b**) CLSM images of MCF-7 cells incubated with PF NPs as a function of incubation time. Red fluorescence was from monomeric phthalocyanine molecules. Green fluorescence showed the generated ROS in situ. (**c**) UV/Vis spectrum and (**d**) fluorescence spectrum of PF NPs in the presence of DSPE-PEG2000 and in an MCF-7 cell suspension. (**e**) Viability of MCF-7 cells incubated with PF NPs in the dark and under laser irradiation (680 nm). Adapted from [206,207] and modified. Copyright (2019) Wiley. Used with permission from Li et al. (2019) [207].

A representative illustration of this concept is provided by peptide–phthalocyanine conjugates that undergo spontaneous self-assembly into nanoparticles in aqueous environments (Figure 16). In this aggregated state, strong π–π interactions within the phthalocyanine cores suppress fluorescence and ROS generation, rendering the system largely photo-inactive under extracellular conditions. However, as demonstrated in Figure 15, cellular internalization induces a gradual recovery of monomeric phthalocyanine fluorescence accompanied by a concomitant increase in intracellular ROS production, consistent with partial aggregate disassembly. Spectroscopic signatures obtained in the presence of amphiphilic components and in cell suspensions further corroborate the restoration of photoactive monomers. Importantly, this biologically triggered activation translates into negligible dark cytotoxicity and pronounced photodynamic efficacy upon 680 nm irradiation, underscoring the potential of supramolecular phthalocyanine assemblies to function as intracellularly activated photosensitizers [206,207].

## 5. Conclusions and Future Perspectives

Conjugation of photosensitizers with biomolecules such as carbohydrates, vitamins, coenzymes, amino acids, peptides, aptamers, proteins, antibodies, and affibodies has emerged as a powerful strategy to overcome the long-recognized limitations of conventional PDT agents. By rational selection of the biomolecular scaffold and precise control over the conjugation site, key physicochemical parameters such as solubility in biocompatible media, lipophilicity, charge distribution, and plasma protein interactions can be systematically tuned. In addition to classical targeting ligands, metabolically relevant vectors such as vitamins and coenzymes provide access to endogenous transport pathways and nutrient uptake mechanisms that are frequently upregulated in malignant cells. Collectively, these design principles enable high-affinity targeting of tumour-associated receptors and microenvironmental cues, translating into improved tumour selectivity, more efficient intracellular trafficking, and better control over dark toxicity. As a result, biomolecule–photosensitizer conjugates frequently display superior photodynamic efficacy across in vitro and in vivo models.

Recent advances in experimental models and readouts provide a framework for interrogating these multi-level mechanisms. Three-dimensional spheroids, organoids, and organ-on-chip systems better reproduce gradients of oxygenation, nutrients, and extracellular matrix density that modulate conjugate uptake, aggregation, and photochemical behaviour. In parallel, in vivo studies increasingly move beyond simple tumour volume measurements toward analyses of pharmacokinetics, biodistribution, and immune modulation, including induction of immunogenic cell death and synergy with checkpoint blockade. Together, these developments indicate that future preclinical characterization of biomolecule–PS conjugates should integrate classical photophysical assays with quantitative studies of supramolecular organization and TME-dependent activation.

Notably, some biomolecule–photosensitizer conjugates operate via mechanisms that extend beyond classical ROS-driven PDT. Trastuzumab–IR700 conjugates exemplify this behaviour, where NIR irradiation induces a photochemical switch from a hydrophilic to a hydrophobic state, promoting aggregation at the cell surface and direct membrane disruption. This oxygen-independent, membrane-active mechanism distinguishes such systems from conventional Type II photosensitizers and highlights the broader therapeutic potential of non-classical PDT pathways.

The evolving understanding of PS aggregation illustrates particularly well how supramolecular control can unlock new therapeutic space. Engineered nanostructured PS aggregates capable of photoinduced symmetry-breaking charge separation can operate predominantly via Type I pathways, generating radical species even under hypoxic conditions that compromise classical Type II photosensitizers. At the same time, albumin-assisted or membrane-driven disassembly of nominally “inactive” aggregates into protein-bound monomers can restore fluorescence and singlet oxygen generation inside cells. Such aggregation-governed reactivity, combined with the possibility of programming the switch between Type I and Type II mechanisms through substitution pattern and biomolecular ligands, positions PS-based conjugates as a model platform for mechanism-tunable PDT.

Looking ahead, several directions appear particularly promising. First, the continued refinement of linker chemistry, stoichiometry of labelling, and site-selective conjugation is likely to reduce heterogeneity and improve the predictability of pharmacokinetics for antibody- and protein-based constructs, including photoimmunotherapy agents. Second, multifunctional designs that combine targeting ligands with oxygen-modulating components, immune adjuvants, or theranostic reporters may help to decouple PDT efficacy from local hypoxia and, at the same time, more effectively harness anti-tumour immunity. Third, deeper integration of NIR and NIR-II photosensitizers, bioluminescence-mediated activation, and environment-responsive probes with biomolecular scaffolds should facilitate treatment of deep-seated or otherwise inaccessible lesions.

From a translational perspective, biomolecule–photosensitizer conjugates offer significant advantages by integrating biological targeting, pharmacokinetic control, and therapeutic mechanisms at the design stage. Conjugation with well-characterized biomolecular ligands enables improved tumour selectivity, reduced off-target toxicity, and more predictable biodistribution, facilitating the transition from preclinical models to clinical application. Importantly, the ability of these conjugates to exploit tumour-specific pathways and, in some cases, oxygen-independent mechanisms expands their clinical relevance, particularly for hypoxic or treatment-resistant tumours. Together, these features position biomolecule–photosensitizer conjugates as highly promising, mechanism-driven platforms for translational PDT.

In this context, the deliberate harnessing of aggregate-state photochemistry is likely to remain a future perspective. By treating biomolecule–photosensitizer conjugates as programmable supramolecular entities that sense, integrate, and respond to the biochemical complexity of the tumour microenvironment, it should be possible to develop PDT modalities that are more versatile, less dependent on oxygen availability, and more readily combined with modern immunotherapies. Such constructs have the potential to transform PDT from a niche local modality into a broadly applicable, mechanism-guided component of precision oncology.

## Figures and Tables

**Table 1:** Therapeutic biomolecules and conjugates targeting major hallmarks of cancer.

Selected Features of Tumour	Biomolecule	Example	Cancer Model	References
Immune evasion	Monoclonal antibodies	Pembrolizumab Nivolumab Atezolizumab	Metastatic melanoma Non-small cell lung cancer	[1,2]
Energy metabolism dysfunction	Oligonucleotides Peptides Vitamins/cofactors	siRNA VDAC1-HK2 Biotin–chlorin e6	Glioblastoma Breast cancer Solid tumours, hypoxic models	[4,5,6]
Immortality	Antisense oligonucleotides Peptide vaccines	Imetelstat GV1001	Myelofibrosis	[7,8,9]
Resistance to proliferation-inhibiting signals	Gene therapy	Gendicine; Advexin	Sarcomas Head and neck squamous cell carcinoma	[10,11]
Ability to metastasize	Monoclonal antibodies Peptides	Abituzumab Andecaliximab	Metastatic colorectal cancer Pancreatic cancer Gastric cancer	[12,13,14,15]
Unrestricted proliferation	Monoclonal antibodies Vitamins (folate ligands)	Trastuzumab Cetuximab Folic acid–PS conjugates	Breast cancer Colorectal cancer Head and neck squamous cell carcinoma FR-overexpressing cancers	[16,17]
Apoptosis escape	Antisense oligonucleotides Recombinant proteins	Oblimersen Dulanermin	Melanoma Chronic lymphocytic leukemia Non-small cell lung cancer	[18,19]
Increased angiogenesis	Monoclonal antibodies Fusion proteins	Bevacizumab Aflibercept	Metastatic colorectal cancer Glioblastoma Non-small cell lung cancer Renal cell carcinoma	[20,21,22]
Inflammation	Monoclonal antibodies	Siltuximab	Multiple myeloma Melanoma	[23]

**Table 2:** Summary of biological activity evaluation for biomolecule–photosensitizer conjugates.

Model Type	Description	Application in Biological Evaluation	References
Targeting, binding models	Analysis of affinity, specificity, receptor binding, and competition assays after conjugation	Verification that the biomolecular ligand retains selectivity for target cells (flow cytometry, microscopy, blocking assays)	[110,111]
Photophysical/photochemical models	Measurement of ROS formation, singlet oxygen quantum yield, photobleaching resistance, and irradiation response	Assessment of whether conjugation preserves efficient ROS generation, photostability, and PDT potency	[101,112]
In vitro cytotoxicity models	Cellular viability assays under light vs. dark conditions; mechanistic studies of apoptosis/necrosis/autophagy	Determination of light-to-dark toxicity ratio, mechanistic pathways, and early therapeutic potential	[110,113]
Subcellular localization models	Confocal imaging, organelle-specific markers, and cell-fractionation studies	Linking intracellular localization (mitochondrial, lysosomal, membrane) to cytotoxic mechanism and conjugate performance	[110,112]
In vivo biodistribution, pharmacokinetic models	Optical imaging, PET/SPECT tracking, tumour retention analysis, clearance studies	Evaluation of selectivity in living organisms, systemic distribution, tumour accumulation, and clearance kinetics	[111]
In vivo efficacy models	Tumour growth inhibition, regression, survival studies, and immune profiling	Determination of therapeutic benefit relative to free PS; assessment of ICD	[101]
Safety, toxicology models	Haematology, liver/kidney function tests, histopathology, immunogenicity analysis	Defining the therapeutic window, off-target effects, systemic toxicity, and phototoxicity profile	[112]

**Table 3:** Examples of carbohydrate–photosensitizer conjugates evaluated in experimental cancer models.

Carbohydrate	Photosensitizer	Model	References
D-glucose	Zn(II) phthalocyanine	Mouse fibrosarcoma and a more advanced and aggressive stage of fibrosarcoma	[125]
	In(III) 5, 10, 15, 20-tetraphenylporphyrin	Human melanoma COLO 679 cells	[130,131]
	Chlorin e6	Mammary carcinoma cells	[115]
D-mannose	5,15-diazaporphyrin	Human breast cancer cells	[132]
	Chlorin e6	Human glioblastoma cells	[133]
D-galactose	Zn(II) phthalocyanine	Colon cancer cells	[134]
	7-hydroxy-6-iodo-3H-phenoxazin-3-one	Human glioblastoma cells	[135]
	BODIPY derivative	Human hepatocellular carcinoma cells	[126]

**Table 4:** Examples of aptamer–photosensitizer conjugates evaluated in experimental cancer models.

Aptamer	Photosensitizer	Model	Reference
AS1411	Chlorin e6	DMBA-induced mammary tumours	[141]
MUC1+Fe_3_O_4_@GO	Chlorin e6	Human breast cancer cells	[142]
AS1411	In(III) phthalocyanine	Human breast cancer cells	[143]
Sgc8	Pyropheophorbide a	Human colorectal carcinoma	[129]

**Table 5:** Examples of amino acid–photosensitizer conjugates evaluated in experimental cancer models.

Amino Acid	Photosensitizer	Model	References
Glycine	Bacteriochlorin (F_2_BGly)	Breast and colon cancer cells	[161]
	BODIPY	Laryngeal carcinoma cells	[154]
Aspartic acid	Chlorin e6	Cervical carcinoma cells Breast adenocarcinoma cells	[158,162]
Arginine	Zn(II) phthalocyanine	Cervical cancer cells	[163]
	Si(IV) phthalocyanine	Cervical, breast, and liver cancer cells	[159]
Cysteine	Cu(II) Pyropheophorbide-a	Murine breast cancer cells Human cervical carcinoma	[164]

**Table 7:** Examples of affibody–photosensitizer conjugates evaluated in experimental cancer models.

Affibody	Photosensitizer	Model	References
HER2-binding affibody	pyropheophorbide-a	Breast cancer	[177]
	IRDye700DX		[178]
EGFR-binding affibody	Temoporfin Chlorin e6 Verteporfin Indocyanine green IRDye800CW	Breast cancer Glioblastoma Head and neck cancer Skin cancer	[179,180,181]
PDGFRβ-binding affibody	IRDye700DX	Tumour blood vessels of colorectal cancer	[182]
		(vascular-targeted PDT)	
PD-L1-binding affibody	ICG	Glioblastoma multiforme brain tumours	[178,183,184,185]
HER3-binding affibody	ICG	HER3-positive MCF7 and LS174T cells	[186]

**Table 8:** Examples of antibody–photosensitizer conjugates evaluated in experimental cancer models.

Antibody	Photosensitizer	Model	References
Trastuzumab	IRDye700DX	Breast cancer	[187,189]
	Chlorin e6	Stomach cancers	[190]
Cetuximab	Chlorin e6	Colorectal cancer	[109,112,191]
	IRDye700DX	Head and neck cancer	
Anti-MIA Ab	Zn(II) PcS_4_	Melanoma	[192]
Anti-PD-L1 Ab	IRDye700DX	Ovarian cancer	[193]
Anti-HER2 Ab	Pyropheophorbide-a	Breast cancer	[194]

**Table 9:** Examples of protein–photosensitizer conjugates evaluated in experimental cancer models.

Protein	Photosensitizer	Model	References
Human serum albumin	Chlorin e6	Human colon cancer	[198,199,200]
	Cyclometalated Ir(III) complex	Human breast cancer	
	BODIPY-like PS	Human liver cancer	
Transferrin	Zn(II) PcN_4_ IR820	Human breast cancer	[196]
Luciferase	Chlorin e6	Human breast cancer “deep-seated tumours”	[197]

## Data Availability

No new data were created or analyzed in this study. Data sharing is not applicable to this article.

## Abbreviations

The following abbreviations are used in this manuscript:

2D	Two dimensional
3D	Three dimensional
ATP	Adenosine Triphosphate
BBB	Blood–brain barrier
BL-PDT	Bioluminescence-Mediated Photodynamic Therapy
BODIPY	4,4-difluoro-4-bora-3a,4a-diaza-s-indacene
CALR	Calreticulin
CCR5	Chemokine Receptor Type 5
CCR7	Chemokine Receptor Type 7
CD	Cluster of Differentiation
CPPs	Cell Penetrating Peptides
CTL	Cytotoxic T Lymphocytes
DAMPs	Damage-Associated Molecular Patterns
DCs	Dendritic Cells
DLI	Drug to Light Interval
ECM	Extracellular Matrix
EGFR	Epidermal Growth Factor Receptor
EPR	Enhanced Permeability and Retention
F_4_BMet	5,10,15,20-tetrakis-[2′,3′,5′,6′-tetrafluoro-4′-methanesulfamoyl)phenyl] bacteriochlorin
FBS	Fetal Bovine Serum
FR	Folate receptor
GLUTs	Glucose Transporter
HCC	Hepatocellular Carcinoma
HER2	Human Epidermal Growth Factor Receptor 2
HMGB1	High-Mobility Group Box 1
HSA	Human Serum Albumin
HSP70	Heat Shock Protein 70
ICD	Immunogenic Cell Death
IDO	Indoleamine 2,3-Dioxygenase
NK	Natural Killer Cells
NIR	Near-Infrared
NIR-II	Second Near-Infrared Window (1000–1700 nm)
NIR-IIa	Sub-window of NIR-II (1300–1400 nm)
OoC	Organ on chip
PA	Pyropheophorbide-a
PDT	Photodynamic Therapy
PIT	Photoimmunotherapy
PpIX	Protoporphyrin IX
PS/PSs	Photosensitizer/Photosensitizers
RNI	Reactive Nitrogen Intermediates
RONS	Reactive Oxygen and Nitrogen Species
ROS	Reactive Oxygen Species
SBCS	Symmetry-Breaking Charge Separation
SELEX	Systematic Evolution of Ligands by Exponential Enrichment
TME	Tumour Microenvironment
